# Multiplexed 3D FRET imaging in deep tissue of live embryos

**DOI:** 10.1038/srep13991

**Published:** 2015-09-21

**Authors:** Ming Zhao, Xiaoyang Wan, Yu Li, Weibin Zhou, Leilei Peng

**Affiliations:** 1 College of Optical Sciences, University of Arizona, 1630 East University Blvd., Tucson, AZ 85721, USA; 2 Department of Pediatrics and Communicable Diseases, University of Michigan, 1150 W. Medical Center Drive, Ann Arbor, MI 48109, USA; 3 Department of Molecular and Cellular Biology, University of Arizona, 1007 E. Lowell St., Tucson, AZ 85721, USA

## Abstract

Current deep tissue microscopy techniques are mostly restricted to intensity mapping of fluorophores, which significantly limit their applications in investigating biochemical processes *in vivo*. We present a deep tissue multiplexed functional imaging method that probes multiple Förster resonant energy transfer (FRET) sensors in live embryos with high spatial resolution. The method simultaneously images fluorescence lifetimes in 3D with multiple excitation lasers. Through quantitative analysis of triple-channel intensity and lifetime images, we demonstrated that Ca^2+^ and cAMP levels of live embryos expressing dual FRET sensors can be monitored simultaneously at microscopic resolution. The method is compatible with a broad range of FRET sensors currently available for probing various cellular biochemical functions. It opens the door to imaging complex cellular circuitries in whole live organisms.

A long sought-after goal in biology is to understand how cellular biochemical processes unfold and interact in their native environments inside living organisms. This problem presents two technical challenges: 1) How to convert cellular biochemical status to signals that can be non-invasively measured and 2) How to read out the signal deep inside a live organism with sufficient spatial resolution. Rapid advances are being made on both fronts. The development of various fluorescent sensors has allowed live imaging of biochemical functions^1^; whereas advances in deep tissue fluorescence microscopy make it possible to visualize intact live organisms in 3D with unprecedented spatial and temporal details^2^,^3^,^4^. Bridging these two fronts will allow us to comprehensively analyze complex cellular machineries of development and diseases at the whole organism level.

Currently most deep tissue microscopy techniques are limited to intensity mapping of fluorescent labels and provide little functional information. Intensity-only imaging techniques can be used in conjunction with small molecule or single fluorescent protein based biosensors^5^,^6^ to probe rapid changes such as calcium level change in neuron activities^7^,^8^, but these biosensors are not ideal for long-term monitoring of live organisms, where concentrations of these sensors as well as the optical property of the surrounding tissue may change over time.

Biosensors based on Förster resonant energy transfer (FRET) between two distinct fluorescent proteins (FPs) allow quantitative characterizations of physiological states^9^ and robust probing of interactions between proteins^10^ in living systems. FRET imaging has therefore long been established as the method of choice for *in vitro* functional imaging of cultured cells. A large selection of FRET sensors are ready to be used for *in vivo* studies of animal models. But to date, FRET has very limited applications *in vivo* because there lacks a fast and robust FRET imaging method in deep tissue. Of the many readout methods for *in vitro* FRET studies, fluorescence lifetime imaging (FLIM) is deemed to be the most robust, because it provides absolute quantification of FRET in single-shot measurement, and is less susceptible to artifacts due to concentration changes, tissue scattering and absorption. Deep tissue fluorescence lifetime imaging (FLIM) with diffuse fluorescence tomography^11^ has been demonstrated for FRET imaging *in vivo*, albeit with low spatial resolution. Projection tomography FLIM^12^ could potentially be a better FLIM method for deep tissue quantitative FRET functional microscopy *in vivo*.

However, the pursuit for a deep-tissue FRET imaging technique should not stop at being able to image a single FRET sensor. As one step further, we report a multiplexed FLIM-FRET deep tissue imaging method that is: (1) compatible with a large selection of FRET sensors, (2) parallel multiplexed on multiple sensors for simultaneously studying complex biochemical interactions, and (3) fast and robust for whole embryo functional microscopy *in vivo*.

The key challenge in multiplexed FLIM-FRET deep tissue imaging is parallel multiplexing. Multiplexed FRET imaging has been performed *in vitro* via various approaches^9^,^13^,^14^,^15^,^16^,^17^. All existing approaches require either sequentially performing multiple imaging exposures by switching excitation sources, which prolongs the acquisition time; or merging two sets of FRET imaging systems into one, which is not affordable for most laboratories. The challenge is even more profound for multiplexed FLIM-FRET, potentially the most robust solution of multiplexed FRET. Multiplexed FLIM-FRET requires lifetime measurements of multiple fluorophores with different excitation wavelengths. In most existing FLIM methods, excitation-multiplexing is achieved by using sophisticated switchable multi-wavelength lasers or time-sharing pulsed lasers^18^,^19^. These approaches, besides further slowing down the already slow imaging acquisition speed of FLIM, add considerably more complexity and cost to the system. Due to cost and speed bottlenecks, multiplexed FLIM-FRET was never attempted *in vitro*, let alone *in vivo* 3D imaging.

We recently developed a parallel excitation FLIM method termed Fourier multiplexed FLIM (FmFLIM) to perform simultaneous fluorescence lifetime measurements on multiple fluorophores with multiple excitation laser lines without switching (See Methods)^20^. Fluorescence signals are separated by both excitation and emission wavelengths into multiple spectral channels, with all excitation-emission (Ex-Em) channels measured in parallel. The method has been successfully applied to simultaneous confocal imaging of multiple fluorescence proteins (FPs)^21^ and cellular FRET study on protein conformation changes^22^. In this paper, we report a technique that combines FmFLIM with scanning laser optical tomography^23^ (SLOT) to perform non-invasive quantitative FRET imaging of multiple FRET sensors in deep tissue and obtain multiplexed 3D functional images of live embryos.

SLOT is a single-beam optical projection tomography^3^ method that is the fluorescence emission analog to single-beam X-ray CT (See Methods, Supplementary Fig. S1). Similar to CT, optical projection tomography can be implemented with multiple beams (wide field illumination) and a multi-element detector (camera)^24^; or with a single scanning beam (focused laser beam), a point detector and simple emission condensing optics^23^. The latter form (SLOT) allows more efficient collection of fluorescence photons in comparison to the wide-field approach. The spatial resolution of SLOT is isotropic in 3D, and is limited by the balance between the waist length and width of the exciting Gaussian beam. Our FmFLIM-SLOT system achieves a spatial resolution of 25 μm (Supplementary Fig. S2) and a depth-of-field of more than 1 mm.

To perform dual FRET imaging in deep tissue, we chose to combine two commonly used FRET pairs: Cyan (CFP)—yellow (Venus) and green (GFP)—red (mCherry). Because GFP and Venus have largely overlapping excitation-emission properties, they were detected in the same Ex-Em channel. These four fluorescence proteins were therefore imaged in three distinct Ex-Em channels: (1) 405-blue channel, which detected photon signals of CFP, (2) 488-green channel, which detected mixed photon signals from Venus and/or GFP excited by the 488 nm laser, and (3) 561-Red channel, which detected photon signals from mCherry excited by the 561 nm laser. Triple-channel lifetime and intensity measurements were performed in parallel. Quantification of dual FRET sensors was achieved by analyzing triple-channel intensity and lifetime images in conjugation. The effectiveness of the multiplexed FLIM-FRET imaging method was demonstrated by simultaneous monitoring of Ca^2+^ and cAMP concentrations with tissue-specifically expressed FRET sensors in transgenic zebrafish embryos (See Methods).

## Results

The FmFLIM-SLOT system consists of two modules (Supplementary Fig. S3a). The FmFLIM module, which performs rapid parallel excitation-multiplexed lifetime measurements on multiple excitation-emission spectral channels (spectral configuration shown in Supplementary Fig. S3b), has been described previously^20^,^21^ (see Methods). The SLOT module scans the focused multi-wavelength laser beam across the sample and performs full-rotation single-beam emission tomography. Fluorescence signals from the sample are collected as 2D projections of hyperspectral fluorescence lifetime decays *S*(*x*, *z*, *θ*; *ω*, *λ*_*x*_, *λ*_*m*_), in which SLOT generates the spatial information (*x*, *z*, *θ*), where θ is the angle of projection, and FmFLIM measures the frequency-domain hyperspectral lifetime decay data as a function of 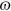 (the modulation frequency of the excitation laser, See Methods). The 3D volumetric hyperspectral lifetime decay data *S*′(*x*, *z*, *y*; *ω*, *λ*_*x*_, *λ*_*m*_) are reconstructed from 180 frames of 2D SLOT projections, taken at an angle interval of Δ*θ* = 2° via the standard filtered-back-projection algorithm. Lifetime analysis on 3D hyperspectral lifetime decay data *S*′(*x*, *z*, *y*; *ω*, *λ*_*x*_, *λ*_*m*_) then yields the hyperspectral intensity *I*(*x*, *z*, *y*; *ω*, *λ*_*x*_, *λ*_*m*_) and lifetime *τ*(*x*, *z*, *y*; *ω*, *λ*_*x*_, *λ*_*m*_) volumes of the sample, which are used for quantitative analysis of FRET sensors.

### Multiplexed 3D FLIM of live embryo

**Figure 1:**
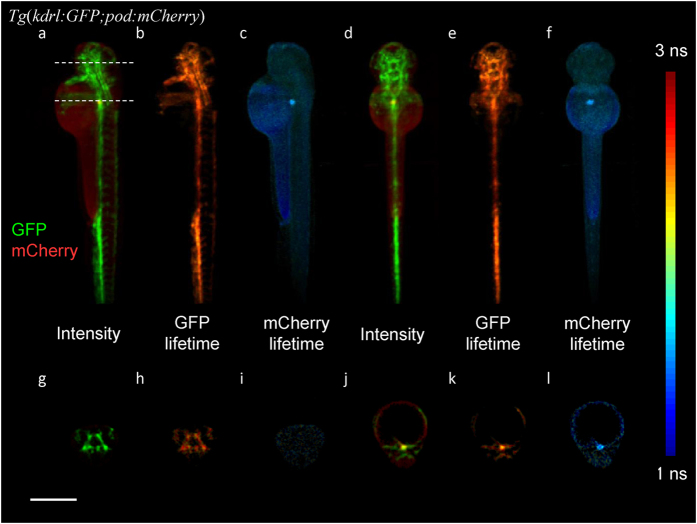
Volumetric fluorescence lifetime images of a *Tg* (*kdrl:GFP;pod:nfsB-mCherry*) zebrafish embryo^25^ at 72 hpf. (**a**) Lateral projection of false color fluorescence intensity with GFP in green (488-green channel) and mCherry in red (561-red channel). (**b**,**c**) Lateral projection image of false color GFP lifetime and mCherry lifetime. Lifetime was rendered as color according to the color index on the right and intensity was rendered as brightness. (**d**–**f**) Dorsal projections of fluorescence intensity, GFP lifetime and mCherry lifetime. (**g**–**i**) Cross section fluorescence intensity, GFP lifetime and mCherry lifetime images of the zebrafish head at the upper dotted line marked in (**a**). (**j**–**l**) Cross section fluorescence intensity, GFP lifetime and mCherry lifetime images of the zebrafish kidney at the lower dotted line marked in (**a**). The color index of all fluorescence lifetime images is shown to the right. Scale bar, 500 μm.

The FmFLIM-SLOT system was applied to *in vivo* 3D FLIM imaging of live transgenic zebrafish embryos and larvae. Figure 1 shows rendered projections and cross sections of 3D dual-channel FLIM images (Supplementary Movie 1) from a double transgenic zebrafish embryo *Tg* (*kdrl:GFP;pod:nfsB-mCherry*)^25^ at 72 hpf (hours post fertilization). These embryos expressed GFP (488-green channel) in endothelial cells and mCherry (561-red channel) in renal glomeruli. In the false color intensity projections (Fig. 1a,d), the vasculature of the embryo is clearly seen throughout the body in green, whereas the kidney is seen at the center of the embryo body in red. GFP lifetime was approximately 2.5 ns throughout the embryo as shown in the intensity weighted false color fluorescence lifetime projections (Fig. 1b,e). mCherry expressing can be clearly separated from background autofluorescence with a lifetime of about 1.7 ns compared to the ~1 ns lifetime of autofluorescence (Fig. 1c,f). Cross sections (Supplementary Movie 2 and Fig. 1g~i) of the embryo show that the vasculature was imaged with isotropic spatial resolution throughout the entire 0.5 mm-wide head.

The FmFLIM-SLOT system is capable of multiplexed 3D FLIM imaging with up to 4 excitation laser lines, which allow 4 spectrally distinct fluorophores to be imaged in parallel. Supplementary Movie 3 presents 4-channel 3D FLIM results from a double transgenic *Tg* (*enpep:GFP;pod:nfsB-mCherry*) zebrafish embryo (72 hpf) with GFP (488-green channel) in kidney tubules, mCherry (561-red channel) in renal glomeruli, Cy5-conjugated dextran (640-deep red channel) in blood vessels via injection, and Syto 41 nuclear stain (Invitrogen, 405-blue channel) in sensory neurons^26^. All intensity and lifetime information were collected simultaneously without switching or time-sharing lasers. With its true parallel multiplexing ability, FmFLIM-SLOT makes multiplexed 3D FLIM as fast and sensitive as single channel FLIM.

### 3D Functional imaging with FRET biosensors

Parallel multiplexed FLIM imaging enables multiplexed 3D functional imaging of entire embryos via FLIM-FRET. Two FRET sensors: GFP-Epac-mCherry (GEpacmC) sensor for monitoring cAMP level^27^, and CFP-D2-cpVenus (CD2V) Calmodulin sensor for monitoring Ca^2+^ level^28^, were selected for probing biochemical conditions in live embryos. The GEpacmC sensor has a single cAMP binding domain, whose binding with cAMP induces a conformation change that increases the distance between GFP and mCherry and causes decrease in FRET efficiency and increase in donor (GFP) lifetime. The CD2V sensor is chosen for its broad Ca^2+^ sensitivity from less than 100 nM up to a few hundred μM. The CD2V sensor consists of two Ca^2+^ binding elements, whose binding with Ca^2+^ leads to conformational changes that pull CFP and Venus closer and cause increase in FRET efficiency between CFP and Venus and decrease in donor (CFP) lifetime. Transgenic zebrafish expressing one of the above sensors in kidney tubules were generated to demonstrate *in vivo* functional imaging with FRET sensors (see Methods). To avoid a high expression level of FRET sensors interfering with the endogenous concentrations of secondary messengers such as cAMP and Ca^2+^, expression of FRET sensors was induced through the Tetracycline-controlled transcriptional activation method and controlled by using an appropriate treatment of doxycycline that did not impose a negative impact on normal zebrafish embryonic development.

### Ca^2+^ imaging with CD2V sensor

**Figure 2:**
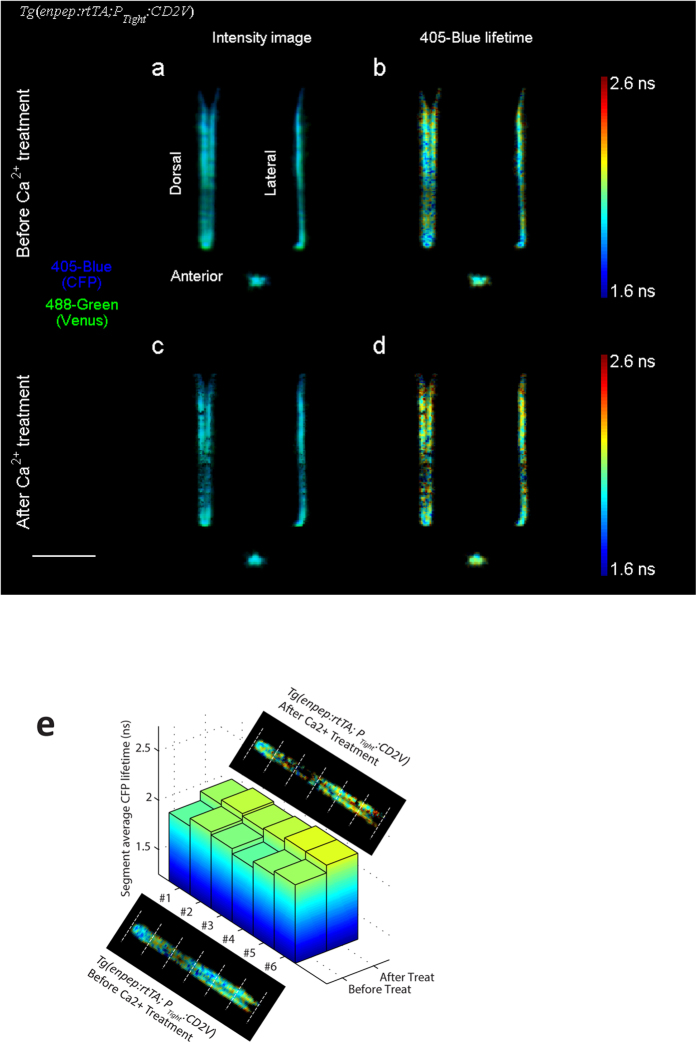
Dorsal, lateral and anterior projections of a 33 hpf *Tg*(*enpep:rtTA; P*_*Tight*_*:CD2V*) zebrafish embryo expressing CD2V sensor in kidney tubules, before and after being treated with 3 mM EGTA, 100 μM BAPTA-AM and 10 μM ionomycin for 2 hours. (**a**,**c**) False color fluorescence intensity projections before and after the treatment, with CFP in blue (405-blue channel) and Venus in green (488-green channel). (**b**,**d**) CFP lifetime varies along the kidney tubule. After the treatment, CFP lifetime increased, on average, by 0.1 ns over the entire kidney tubule. (**e**) Average CFP lifetimes of 6 segments of kidney tubules divided equally, before and after the Ca^2+^ treatment. Despite significant variations in average CFP lifetimes between segments, CFP lifetimes in all segments increased after the treatment. Scale bar, 500 μm.

Double transgenic zebrafish embryos *Tg*(*enpep:rtTA; P*_*Tight*_*:CD2V*) with doxycycline-induced CD2V expression in kidney tubules were imaged at approximately 33 hpf (Fig. 2a,b and Supplementary Movie 4). The fluorescence lifetime of CFP (Fig. 2b, 1.98 ns on average) exhibits large spatial variation in tubule cells, which may suggest variations in Ca^2+^ concentrations (See Discussion). The CFP lifetime of CD2V sensor in kidney tubules was shorter compared to the lifetime of purified CFP in solution (2.6 ns, measured by FmFLIM) due to two reasons: First, for any FRET sensors, there is likely to be a baseline FRET between donor and acceptor molecules, leading to a decreased donor lifetime comparing to lifetime of free donor molecules; Second, binding of Ca^2+^ leads to an increase in FRET efficiency of CD2V sensors, and further decreases CFP lifetime.

The embryo was then subjected to a treatment that reduces the Ca^2+^ level in the entire embryo. FRET efficiency of CD2V sensor decreases when Ca^2+^ ions are removed from sensor binding sites, thus, an increase in CFP lifetime, detected through the 405-blue channel, was expected after the Ca^2+^ decreasing treatment. After being treated with calcium chelators EGTA (3 mM), BAPTA-AM (100 μM) and ionophore ionomycin (10 μM) for 2 hours, the embryo was scanned again (Fig. 2c,d). CFP fluorescence lifetime (Fig. 2d) on average increased by 0.1 ns to 2.08 ns as expected (Supplementary Fig. S4a). The 488-green channel photon signal, which collected direct excitation-emission from Venus, was detected in parallel with the 405-blue channel. Because the signal in the 488-green channel was purely from the self-excitation of Venus (acceptor of the CD2V sensor) and was unrelated to the FRET process, the 488-green channel was used as a bystander control of the FLIM-FRET measurements. As expected, the lifetime in the 488-green channel was unchanged at 2.98 ns after the treatment (Supplementary Fig. S4b). Although CFP lifetime had significant spatial inhomogeneity, a spatial correlation analysis shows that average CFP lifetime increased in all segments along the tubule after the Ca^2+^ treatment (Fig. 2e), indicating that the treatment increased Ca^2+^ levels evenly. Furthermore, CFP lifetime increases were consistently observed in 5 embryos between 30~36 hpf (Supplementary Fig. S5a, CFP lifetime changed by 0.10 ± 0.02 ns). In the meantime, Venus lifetime detected through the 488-green channel showed no significant changes in these embryos after treatments (Supplementary Fig. S5b).

We also observed that the Ca^2+^ treatment lost effect in embryos older than 36 hpf (Supplementary Fig. S6, donor CFP lifetime changed by −0.003 ± 0.039 ns), which may due to decreases in Ca^2+^ surges and transients (See discussion). In all experiments, treatment drugs were rinsed off after the second imaging scan and all embryos developed normally afterwards.

### cAMP imaging with GEpacmC sensor

**Figure 3:**
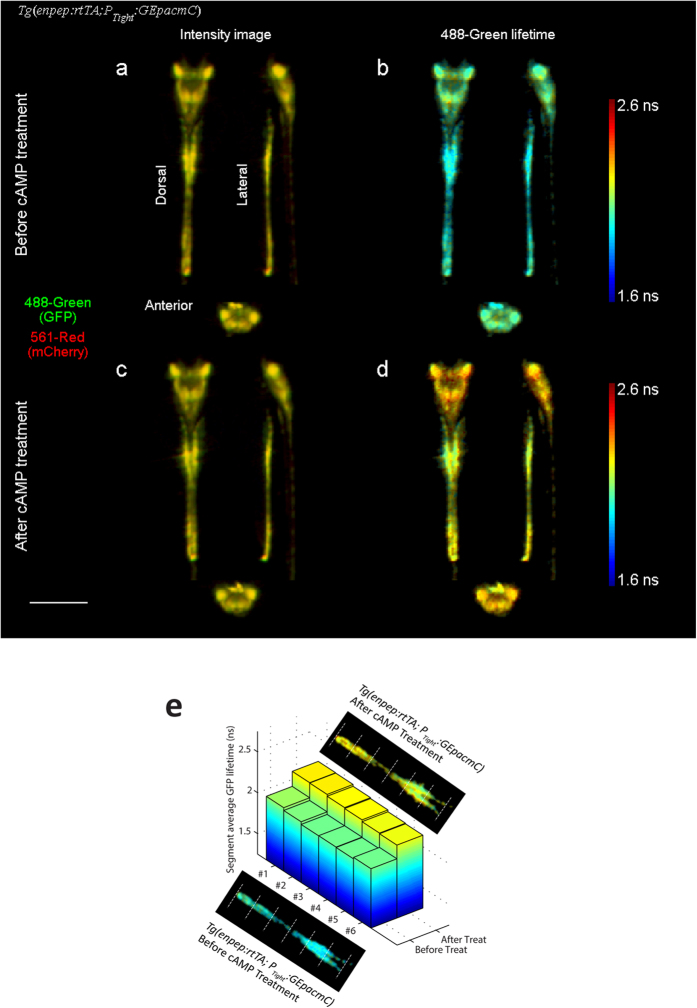
Dorsal, lateral and anterior projections of a 48 hpf *Tg*(*enpep:rtTA; P*_*Tight*_*:GEpacmC*) zebrafish embryo expressing GEpacmC sensor in kidney tubules and neurons, before and after being treated with 100 μM forskolin and 400 μM IBMX for 2 hours. (**a**,**c**) False color fluorescence intensity projections before and after the treatment, with GFP in green (488-green channel) and mCherry in red (561-red channel). (**b**,**d**) GFP lifetime before and after treatment, showing tissue specific variation, which may indicate variations in cAMP level. After the treatment, GFP lifetime increased by 0.2 ns evenly over all tissue types. (**e**) Average GFP lifetimes in 6 segments of kidney tubules divided equally along the tubule, before and after the cAMP treatment. All segments exhibited GFP lifetime increase after the treatment. Scale bar, 500 μm.

Similar FLIM-FRET studies were performed on double transgenic zebrafish embryos *Tg*(*enpep:rtTA; P*_*Tight*_*:GEpacmC*) with induced expression of GEpacmC sensor^27^. The *enpep:rtTA* transgenic line appeared to have neuronal in addition to pronephric expression in a small fraction of embryos, as seen in an embryo imaged at about 48 hpf (Fig. 3a,b and Supplementary Movie 5). GFP lifetime, measured through the 488-green channel, was longer in neurons (average 2.06 ns) compared to lifetime measured in kidney tubules (average 1.98 ns). Slight spatial variations in GFP lifetime along the kidney tubules were observed. GFP lifetime in all tissues expressing GEpacmC sensors were lower than the free GFP lifetime (2.5 ns) measured from the *Tg* (*kdrl:GFP*) zebrafish embryos. The decrease in GFP lifetime comparing to free GFP lifetime was mainly caused by the baseline FRET between donor and acceptor molecules as mentioned previously. Since the GEpacmC sensor decreases its FRET efficiency and increases the donor (GFP) lifetime upon binding to cAMP, the spatial pattern of GFP lifetimes may suggest that kidney tubules exhibit a lower concentration of cAMP than neurons. Variations in cAMP concentration among different tissue types are unsurprising and yet unreported before, because such phenomenon is only observable by a deep-tissue FRET imaging technique that can penetrate to the center of embryo and has a sufficient spatial resolution to identify different organs or structures.

The same zebrafish embryo was then subjected to pharmacological stimuli to increase cellular cAMP concentration (100 μM adenylyl cyclase activator forskolin and 400 μM phosphodiesterase inhibitor IBMX). The embryo was rescanned after 2 hours of the cAMP treatment (Fig. 3c,d). As expected, GFP lifetime increased after the treatment. The amount of increase was similar across the embryo regardless the pre-existing spatial variation in GFP lifetime (Supplementary Fig. S7a): the average GFP lifetime increased to 2.18 ns in kidney tubules and 2.26 ns in neurons. The lifetime of the 561-red channel, which observed the direct excitation of mCherry and served as a bystander control, remained the same (Supplementary Fig. 7b). Spatial correlation analysis shows that despite variations in average lifetimes, all segments of tubules exhibited consistent GFP lifetime increases after the treatment (Fig. 3e).

Effects of the cAMP treatment were consistently observed for embryos at different ages from 30 to 60 hpf. Supplementary Fig. S8 shows cAMP treatment results of multiple embryos between 40 and 48 hpf. These embryos exhibited GFP lifetime increase at 0.15 ± 0.05 ns after treatments, while the lifetime in bystander 561-red channel was unchanged (0.014 ± 0.017 ns). With a prompt washing after the second imaging scan, development of the embryos were not affected by the cAMP treatment.

Effects of the Ca^2+^ treatment on GEpacmC sensor (Supplementary Fig. S9, GFP lifetime change 0.002 ± 0.018 ns; mCherry lifetime change 0.017 ± 0.018 ns) and the cAMP treatment on CD2V sensor (Supplementary Fig. S10, CFP lifetime change −0.003 ± 0.012 ns; Venus lifetime change −0.020 ± 0.005 ns) were also tested in embryos. Neither of these treatments affected donor lifetimes of off-target FRET sensors.

### Multiplexed 3D FLIM-FRET imaging

Hyperspectral volumetric lifetime images from FmFLIM-SLOT allow functional imaging of multiple FRET sensors. As a demonstration, we used three Ex-Em channels (405-blue, 488-green and 561-red) to simultaneously detect four FPs in CD2V and GEpacmC sensors, with the 405-blue channel probing CFP, the 561-red channel probing the direct-excitation of mCherry, and the 488-green channel collecting signals from both GFP and Venus. The average fluorescence lifetime from the 488-green channel is the intensity-weighted average lifetime of GFP and Venus, which is affected by both GFP-mCherry FRET and the direct excitation of Venus. Since the lifetime of directly excited Venus is not related to any FRET process and remains constant, any lifetime change in the 488-green channel is caused by changes in GFP lifetime and therefore cAMP concentration changes.

**Figure 4:**
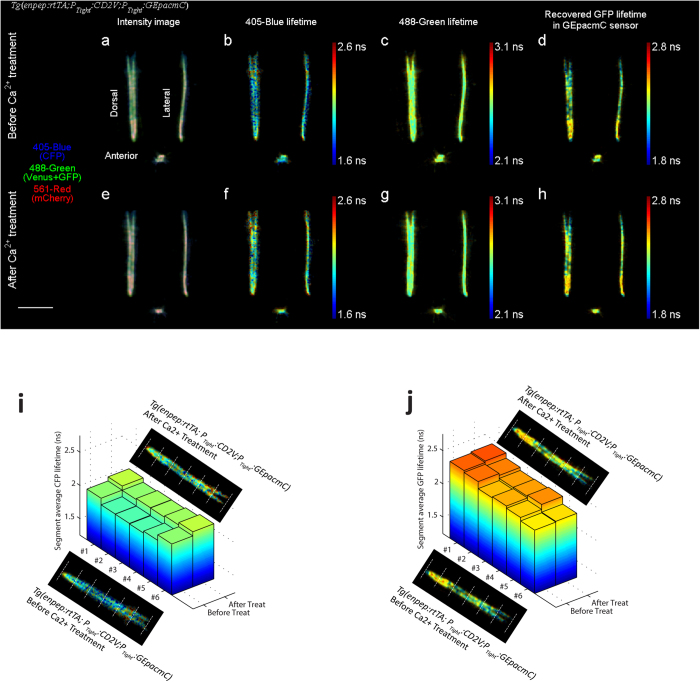
Dorsal, lateral and anterior projections of a 33 hpf *Tg*(*enpep:rtTA; P*_*Tight*_*:CD2V;P*_*Tight*_*:GEpacmC*) zebrafish embryo expressing both CD2V sensor and GEpacmC sensor, before and after being treated with 3 mM EGTA, 100 μM BAPTA-AM and 10 μM ionomycin for 2 hours to decrease cellular Ca^2+^ level. (**a**,**e**) False color fluorescence intensity projections before and after treatment, with 405-blue channel in blue, 488-green channel in green and 561-red channel in red. (**b**,**f**) 405-blue channel fluorescence lifetime (CFP lifetime) increased by 0.10 ns after treatment. (**c**,**g**) 488-green channel fluorescence lifetime (intensity weighted average of Venus and GFP lifetimes) was not affected by treatment. (**d**,**h**) Recovered GFP lifetime in GEpacmC sensor by the triple-channel intensity lifetime analysis. GFP lifetime was not changed by the treatment. (**i**) Average CFP lifetimes in 6 segments of kidney tubules divided equally, before and after the Ca^2+^ treatment. Despite significant variations in average CFP lifetimes between segments, CFP lifetimes in all segments increased after the treatment. (**j**) Average recovered GFP lifetimes in 6 tubule segments, before and after the Ca^2+^ treatment. GFP lifetime showed pronounced spatial variation. This spatial pattern of GFP lifetimes was not significantly altered by the Ca^2+^ treatment. Scale bar, 500 μm.

The multiplexed functional imaging scheme was demonstrated on triple transgenic zebrafish embryos *Tg*(*enpep:rtTA; P*_*Tight*_*:CD2V; P*_*Tight*_*:GEpacmC*) expressing both the CD2V and GEpacmC sensors in kidney tubules (See Methods). Intensities and lifetimes of three Ex-Em channels were simultaneously imaged for a whole embryo (Fig. 4a–c and Supplementary Movie 6) at approximately 33 hpf. The embryo was then subjected to the Ca^2+^ treatment and imaged again (Fig. 4e–g). CFP lifetime (405-blue channel) taken after the treatment increased evenly by 0.10 ns despite a spatially heterogeneous lifetime distribution, indicating the Ca^2+^ level decreased universally as expected (Supplementary Fig. S11a). The average fluorescence lifetime in the 488-green channel (Supplementary Fig. S11b) and in the 561-red channel (Supplementary Fig. S11c) remained unchanged.

**Figure 5:**
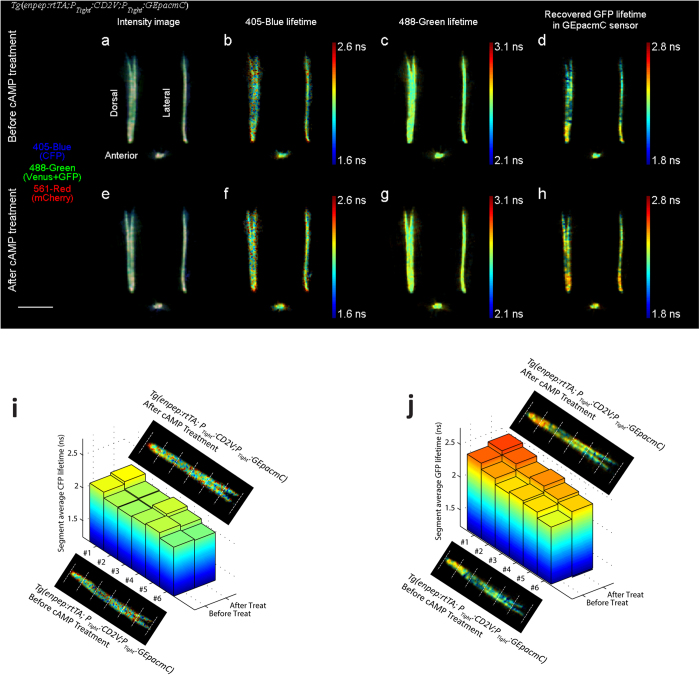
Dorsal, lateral and anterior projections of a 48 hpf *Tg*(*enpep:rtTA; P*_*Tight*_*:CD2V;P*_*Tight*_*:GEpacmC*) zebrafish embryo expressing both CD2V sensor and GEpacmC sensor, before and after being treated with 100 μM forskolin and 400 μM IBMX for 2 hours to increase cellular cAMP level. (**a**,**e**) False color fluorescence intensity projections before and after treatment, with 405-blue channel in blue, 488-green channel in green and 561-red channel in red. (**b**,**f**) 405-blue channel fluorescence lifetime (CFP lifetime) remained the same after treatment. (**c**,**g**) 488-green channel fluorescence lifetime (intensity weighted average of Venus and GFP lifetimes) was slightly increased by the treatment. (**d**,**h**) Recovered GFP lifetime by the triple-channel intensity lifetime analysis. GFP lifetime increased by 0.1 ns after the cAMP treatment. (**i**) Average CFP lifetimes in 6 segments of the kidney tubule divided equally, before and after the cAMP treatment. CFP lifetimes in different segments showed significant spatial variation, and were not significantly affected by the cAMP treatment. (**j**) Average recovered GFP lifetimes in 6 tubule segments, before and after the cAMP treatment. Although GFP lifetime had significant segment-to-segment variation, they all exhibited an increase after the cAMP treatment. Scale bar, 500 μm.

The effect of cAMP treatment on these dual FRET sensor expressing embryos *Tg*(*enpep:rtTA; P*_*Tight*_*:CD2V; P*_*Tight*_*:GEpacmC*) was investigated in a similar fashion. A baseline image of an embryo was taken at about 48 hpf (Fig. 5a–c and Supplementary Movie 7). The embryo was then subjected to the cAMP treatment, and rescanned 2 hours after treatment (Fig. 5e–g). CFP lifetime in the 405-blue channel remained the same after treatment, indicating the Ca^2+^ level was not affected by the cAMP stimulus treatment (Supplementary Fig. S12a). The average lifetime in the 488-green channel increased by 0.037 ns (Supplementary Fig. S12b), suggesting an increase in GFP lifetime and thus an increase in the cAMP level. The mCherry lifetime in the 561-red channel (bystander) remained the same (Supplementary Fig. S12c).

### Lifetime analysis of multiplexed FLIM-FRET

The average lifetime of the 488-green channel signal is the intensity weighted average of GFP and Venus lifetimes, and serves only a qualitative indicator for changes in FRET efficiency of GEpacmC sensor. We therefore used a triple-channel combined intensity-lifetime analysis method to quantitatively analyze dual FRET sensors. The method recovers the GFP lifetime by using intensity and lifetime information on all three Ex-Em channels together (Supplementary Notes). Briefly, the method is based on the fact that donors and acceptors in single-link FRET sensor pair are expressed at 1:1 concentration ratio. Using fluorescence intensity images of mCherry from the 561-red channel or CFP from the 405-blue channel, the intensity ratio of Venus vs. GFP in the 488-green channel can be calculated. Since the lifetime of Venus is constant and can be predetermined by measuring single CD2V sensor embryos, once the intensity ratio of Venus vs. GFP is known, the GFP lifetime can be extracted from the average lifetime in the 488-green channel.

Using the above analysis, the GFP lifetime of GEpacmC sensor was recovered from triple-channel FLIM data of *Tg*(*Enpep:rtTA; P*_*Tight*_*:CD2V; P*_*Tight*_*:GEpacmC*) embryo (Figs 4d,h and 5d,h). Variations in GFP lifetime along the kidney tubule were observed in a spatial pattern similar to the *Tg*(*enpep:rtTA; P*_*Tight*_*:GEpacmC*) embryo in Fig. 3: all three embryos showed longer GFP lifetime in distal tubules, which may indicate distal tubules have a higher cAMP level.

The analysis also revealed that after the Ca^2+^ treatment, the average GFP lifetime of *Tg*(*Enpep:rtTA; P*_*Tight*_*:CD2V; P*_*Tight*_*:GEpacmC*) embryo remained the same, confirming that the cAMP level was undisturbed by the off-target treatment (Supplementary Fig. S13a). On the other hand, after the cAMP treatment, the average GFP lifetime of *Tg*(*enpep:rtTA; P*_*Tight*_*:CD2V; P*_*Tight*_*:GEpacmC*) embryo increased from 2.26 ns (Fig. 5d) to 2.36 ns (Fig. 5h and Supplementary Fig. S13b). These positive and negative results prove that the analysis can truthfully quantify the GEpacmC sensor FRET out of two co-expressed FRET sensors despite a large spectral overlap between two sensors.

Ca^2+^ and cAMP treatment results were consistent for multiple *Tg*(*Enpep:rtTA; P*_*Tight*_*:CD2V; P*_*Tight*_*:GEpacmC*) embryos. The Ca^2+^ treatment caused CFP lifetime increasing by 0.08 ± 0.01 ns in 5 embryos, and changes in recovered GFP lifetimes were near zero (0.016 ± 0.010 ns) after the Ca^2+^ treatment (Supplementary Fig. S14). The cAMP treatment caused GFP lifetime increasing by 0.06 ± 0.02 ns, whereas CFP lifetime in the 405-blue channel was not affected by the treatment (0.004 ± 0.014 ns, Supplementary Fig. S15)

Spatial correlation analysis was also applied to these multiplexed FRET imaging results. In the Ca^2+^ treated *Tg*(*Enpep:rtTA; P*_*Tight*_*:CD2V; P*_*Tight*_*:GEpacmC*) embryo, average CFP lifetimes of tubule segments (Fig. 4i) all increased after the treatment regardless of the initial spatial CFP lifetime variation, and recovered GFP lifetimes of all segments (Fig. 4j) were not significantly affected by the treatment. In the cAMP treated embryo, average CFP lifetimes of all segments were not affected (Fig. 5i), and the average recovered GFP lifetimes showed consistent increase along tubules (Fig. 5j).

## Discussions

### FmFLIM-FRET measurement in live embryos

Combined with advanced genetic manipulation, FmFLIM-SLOT enables investigations of multiple physiological states or protein interactions in specific tissue types. We observed that Ca^2+^ level varied along kidney tubules in zebrafish embryos. Growing evidence shows that both intracellular and intercellular Ca^2+^ signaling plays key roles for developing embryos in the process of establishing complexity^29^. Ca^2+^ signaling activities depend highly on the specific organ and/or the development stage and can have various forms including waves, transients and gradients^29^,^30^,^31^. Recently, heterogeneous basal calcium level and spontaneous calcium oscillations within kidney tubules were also reported in live adult rats^32^. Therefore, the observed Ca^2+^ level variation along kidney tubules is well expected.

In this study, we demonstrated that FmFLIM-SLOT can observe and track the spatial pattern of Ca^2+^ levels over a long period of time, during which the effect of Ca^2+^ treatment was successfully measured despite pre-existed spatial variation. Spatial correlation analysis of treatment image data is the key to quantify these time-spatial functional images. Here we performed a simple spatial correlation analysis that divided the tubules into short vertical sections and compared segment-averaged lifetimes before and after treatment (Fig. 2e). As each segment consists hundreds of pixels, lifetime measurement uncertainty plays a negligible role in influencing the average lifetime of the segment. The facts that the spatial variation still exist after aggressive averaging and lifetime of all segments changed in concert strongly suggest that there are underlying mechanisms governing the spatial pattern of Ca^2+^ level in tubules, which will be subjects of future studies.

Aiming at decreasing Ca^2+^ concentrations within kidney tubules, we chose a Ca^2+^ treatment composed of EGTA, BAPTA-AM and ionomycin. Interestingly, no detectable change of CFP lifetime was observed without BAPTA-AM (data not shown). It is well known that BAPTA-AM binds and releases Ca^2+^ ion much faster than EGTA^33^ and can be used to down regulates Ca^2+^ surges during zebrafish development^34^. The critical role of BAPTA-AM indicates that this treatment is probably acting on fast Ca^2+^ surges and transients. We observed that this treatment was not effective on embryos older than 36 hpf. This age dependence further suggests that the treatment loses effect probably because the Ca^2+^ surges subsides once the tubules mature, as similar phenomenon were observed in other major organs^29^.

We also observed cAMP level variations along kidney tubules between 40~48 hpf within live zebrafish embryos. Induced cAMP oscillations on the scale of several minutes within human embryonic kidney cells have been reported^35^. But, unlike Ca^2+^ signaling, much less is known regarding the role of cAMP during development, probably due to the fact that live cAMP readout of embryos requires deep tissue FRET imaging. We found GFP lifetimes were consistently higher in distal tubules of all embryos expressing the GEpacmC sensor. The observation strongly indicates that the cAMP level may be higher in distal tubules than in proximal tubules. Through spatial correlation analysis, we found GFP lifetime evenly increased within all segments of tubules after the cAMP treatment, as demonstrated in Fig. 3e, indicating that the treatment elicits universal response regardless pre-existing cAMP level variation in tubule segments.

The observed response to cAMP treatment in double sensor *Tg*(*enpep:rtTA; P*_*Tight*_*:CD2V; P*_*Tight*_*:GEpacmC*) zebrafish embryos (0.06 ± 0.02 ns) was much smaller than the response in single sensor *Tg*(*enpep:rtTA;P*_*Tight*_*:GEpacmC*) embryos (0.15 ± 0.05 ns). This discrepancy may be caused by different GEpacmC expression levels in these embryos. In double sensor expressing *Tg*(*enpep:rtTA; P*_*Tight*_*:CD2V; P*_*Tight*_*:GEpacmC*) embryo, the same promoter was used for expressing two kinds of sensors, thus *Tg*(*enpep:rtTA; P*_*Tight*_*:CD2V; P*_*Tight*_*:GEpacmC*) embryos had a lower expressing level in each sensor compared to *Tg*(*enpep:rtTA; P*_*Tight*_*:CD2V*) or *Tg*(*enpep:rtTA; P*_*Tight*_*:GEpacmC*) embryos. The reduction in expression levels of double sensors affected GEpacmC the most, whose levels in *Tg*(*enpep:rtTA; P*_*Tight*_*:CD2V; P*_*Tight*_*:GEpacmC*) embryos were typically 1/5-1/3 of the levels in single sensor *Tg*(*enpep:rtTA;P*_*Tight*_*:GEpacmC*) embryos. Depending on the specific cell type, the basal concentration of cAMP varies from a few hundred nM to a few μM^36^, which is comparable with the level of GEpacmC in weakly expressing cases. A low GEpacmC expression level may result in a higher percentage of sensor molecules already binding to cAMP, leaving less free GEpacmC sensors to react with cAMP generated by the treatment, and therefore a smaller change in GFP lifetime upon treatment. Similar effects were also observed in our previous live cell cAMP sensor studies (data not shown)^37^. This expression-level dependent response implies that a tight control on the expressing level is required for future quantitative cAMP imaging studies, which is one of the reasons that this study chose to use inducible transgenic expression in embryos, for it allows control over the sensor expression level.

### Fluorescence lifetime accuracy of FmFLIM-SLOT

The fluorescence lifetime accuracy of the FmFLIM method had been previously tested in confocal FLIM applications^21^, which showed that, like other FLIM techniques, the lifetime accuracy of FmFLIM was determined by the shot-noise of fluorescence photons. With a typical signal level of 10,000 photons per pixel, 10% accuracy in lifetime measurement can be achieved. In projection images obtained by SLOT, the lifetime accuracy is subjected to the same shot-noise limit, but the typical photon number per voxel is higher.

X-ray CT research had proved that in all shot-noise limited tomography imaging experiments, the signal-to-noise ratio in a reconstructed voxel depends on the number of detected photons summed over all projection angles that come from the voxel, and the reconstruction process does not significantly add uncertainties to the measurements^38^. The lifetime accuracy of FmFLIM-SLOT is better than that of a single projection image due to this photon summing effect of tomography. As an example, in *Tg*(*kdrl:GFP*) embryos, the number of fluorescent photons per voxel typically is 4 times of the number of photons per pixel detected in a single 2D projection image, therefore fluorescence lifetime calculated from the reconstructed volumetric data (2.56 ± 0.14 ns) is 2 times more accurate than lifetime calculated from one projection image (2.56 ± 0.27 ns) (Supplementary Fig. S16).

The majority of lifetime changes seen in this study are smaller than the single voxel lifetime accuracy, which is a common challenge in analyzing live FLIM imaging results. Fortunately, FLIM provides lifetime-intensity dual modality images. Intensity images, in combination with targeted labeling, provide valuable structure information about the specimen, and are used to guide the statistical analysis of lifetime image. For example, intensity 3D image in Fig. 3 clearly shows that both the kidney tubules and neurons were expressing GEpacmC sensors. The 3D structural information of whole embryo allowed us to separate lifetime data of two tissue types and came to the conclusion that the GFP lifetime, on average, is higher in neurons by 0.08 ns, a small but statistically valid difference.

Similarly, in kidney tubules, the wide distribution of CFP or GFP lifetimes in CD2V or GEpacmC sensors was caused by two factors: the natural spatial variation of Ca^2+^ or cAMP level in tubules and the lifetime measurement uncertainty. To detangle these two factors, spatial correlation analysis of tubules segments was applied. The analysis shows that average donor lifetimes of both GEpacmC and CD2V sensors varied between segments, and lifetimes in all segments raised or remained the same in concert. The analysis utilized structural information about tubules, obtained from intensity image, to statistically analyze lifetime data. The approach allowed measuring small lifetime changes in the present of large spatial variation and measurement uncertainty in lifetimes.

### Applications of FmFLIM-SLOT

The advances in developmental and system biology call for functional imaging techniques that can provide spatial and temporal information on biochemical functions and interactions inside living organisms. While high resolution structural imaging of live organisms has become common practice, reports on functional imaging in live organisms have been scarce with the exception of calcium imaging. FmFLIM-SLOT offers a versatile multiplexed functional imaging method that is compatible with a wide range of FRET sensors for various biochemical functions. In this paper, we demonstrated the technique through volumetric multiplexed FLIM-FRET imaging of live zebrafish embryos expressing two FRET sensors. Results showed that two FRET sensors, detecting Ca^2+^ and cAMP levels respectively, could be reliably read out in parallel at high spatial resolution.

The technique is compatible with FRET sensors with large spectral overlap, and thus can make use of most existing FRET sensors. The full multiplexing capability of FmFLIM-SLOT was yet to be realized, because at present high-quality FRET sensors are limited in the visible spectrum. Should FRET sensors with infrared FP become available, the current FmFLIM-SLOT system could perform functional imaging with three or more FRET sensors in parallel. While the moderate 25 μm spatial resolution of the current system is not sufficient for discerning single cells, it is adequate to resolve intra-organ spatial structure and heterogeneity of biological functions inside live animals.

FLIM is well suited for long-term FRET studies because it is robust against changes in expression level, tissue scattering and absorption, which make ratiometric FRET imaging unreliable in long-term studies. In this study, results from by-stander control channel demonstrated that the FmFLIM-SLOT system is highly stable and reliable over long time operations. The current FmFLIM-SLOT system scans a whole embryo within 12 minutes (3.5×2 × 2 mm^3^). The scanning time could reduce to 3~5 minutes if the region of interest is cut down to a specific organ. The speed is sufficient for tracking long-term changes during development or disease progression.

We further envision that FmFLIM-SLOT could be combined with multi-color light-sheet microscopy^3^,^4^,^39^ to comprehensively observe both fast dynamics and slow developments of biochemical functions in animals. Key optical elements of both imaging modalities are identical and can be shared. Combining these two modalities will allow measuring fast biochemical dynamics at high spatial and temporal resolution through ratiometric FRET light-sheet microscopy, and in the meantime monitoring long-term changes through FmFLIM-SLOT. The marriage of these two techniques will provide unprecedented opportunities in studying complex cellular machineries in whole organism.

## Methods

### Imaging experiments and data processing

The FmFLIM-SLOT system combines the multi-excitation frequency sweeping lifetime measurement of FmFLIM with volumetric imaging of SLOT (Supplementary Fig. S3a).

#### Parallel Ex-Em channel measurements with FmFLIM

FmFLIM simultaneously measures frequency domain fluorescence lifetimes at multiple excitation wavelengths through the principle of Fourier transform fluorescence lifetime spectroscopy^20^,^21^. Instead of separating different laser lines in time by switching lasers on and off, FmFLIM separates excitation laser lines by imprinting unique modulation frequencies onto each laser line so that laser lines of different wavelengths can be distinguished in the frequency domain.

The frequency imprinting is done by a Michelson interferometer with a spinning polygon mirror (48 facets, 55,000 rpm, 2.5 inch diameter) optical delay arm, which generates a fast scanning optical path difference. When multiple excitation lasers (at 405, 488, 561 and 640 nm wavelengths) are modulated by the interferometer, each laser line is modulated at a unique wavelength-dependent instantaneous frequency *f* = *v*/*λ*, where *v* is the instantaneous optical delay scan speed of the interferometer (Supplementary Fig. S17). When the modulated multi-line laser is used to excite a fluorescent sample, fluorescence photons excited by a particular laser line are imprinted with the same unique instantaneous frequency as the excitation laser, and photon signals associated with each excitation line can be read out by radio frequency (RF) down-mixing at a series of matching frequencies. As signals of multiple excitation channels have distinct modulation frequencies and do not interfere with each other during RF down-mixing, multiple excitation channels can be active and detected at the same time through the principle of Fourier (modulation frequency) multiplexing.

In the FmFLIM system, fluorescence photons excited by multiple laser lines are further split by their emission wavelengths and detected by multiple detectors simultaneously. With Fourier excitation multiplexing and multiple emission channels, the FmFLIM system is capable to differentiate photon signals by both excitation and emission spectral properties and detect all excitation-emission (Ex-Em) channels in parallel. The current FmFLIM system is capable of simultaneous measurements on 4 × 4 Ex-Em channels (spectral configuration shown in Supplementary Fig. S3b), suitable for most common fluorescent proteins and dyes in the visible range.

#### Multi-channel lifetime measurements in FmFLIM

The fluorescence lifetime of each excitation-emission channel is measured by the frequency domain lifetime method. In addition to Fourier Ex-Em multiplexing, the FmFLIM system is also capable of performing lifetime measurements at multiple frequency points over a continuous frequency span from 10 MHz to a maximum frequency of 120–200 MHz depending on the laser wavelength. This unique feature is brought on by the polygon-mirror based optical delay line in the interferometer, whose delay scan speed varies linearly from −94 m/s to 94 m/s at a 44 kHz repetition rate. At the output of the interferometer, the laser lines are modulated into round trip frequency sweeps with maximum frequencies ranging between 120 and 200 MHz (corresponding to 640 nm and 405 nm laser line, respectively) within 23 μs (Supplementary Fig. S17). The frequency-sweeping modulations on four laser lines allow nanosecond fluorescence lifetime measurements at a rate of 44,000 measurements/second.

Fluorescence lifetime information on all Ex-Em channels is obtained in parallel through an analog-digital hybrid data analysis method as previously described^21^ (Supplementary Fig. S18). In short, to generate a lifetime measurement signal at a given Ex-Em wavelengths (*λ*_*x*_, *λ*_*m*_) combination, the photon signal from PMT at emission wavelength *λ*_*m*_ and the signal from a photodiode that monitors the frequency modulation of laser line *λ*_*x*_ are sent to an analog RF mixer (Mini-Circuits ZX05-1L-S+) followed by a low pass filtered (Mini-Circuits BLP-10.7+) (Supplementary Fig. S18b). The resulting signal’s carrier frequency is down-mixed to 200~300 kHz. In the *λ*_*m*_ PMT, photon signals excited by laser lines other than *λ*_*x*_ are removed during the down-mix and filtering process. Only signal excited by laser line *λ*_*x*_ contributes to the down-mixed signal, which contains the fluorescence decay information


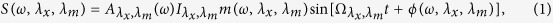


where *ω* is the instantaneous modulation frequency of the laser line *λ*_*x*_, 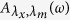 is the RF response characteristics of the electrical circuits in the Ex-Em channel, 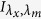 is the intensity of the signal, *m* and *ϕ* are the modulation and phase of the fluorescence lifetime frequency responses of the sample, and 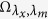 is the carrier frequency of the down-mixed signal set by the heterodyne down-mixing. Down-mixed signals at multiple Ex-Em channels are recorded simultaneously at 2 MHz sampling rate by a multi-channel high-speed digitizer equipped with an FPGA signal processor (NI 5752 & 7962). 44 data points per channel per pixel are recorded to cover the round trip frequency sweep. All data acquisition, hardware control and image scanning are performed with a custom software written in LabVIEW (National Instruments).

The detection system’s complex RF response 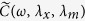, characterized by 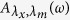 and 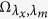, can be pre-calibrated by fluorescence lifetime standards with known single exponential decay lifetime,


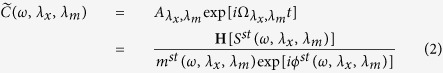


where **H** is the Hilbert transform, *m*^*st*^ and *ϕ*^*st*^ are modulation and phase responses of the lifetime standard calculated from the known single exponential lifetime of the standard. The calibrated system RF response can then be removed from the decay frequency response of the sample


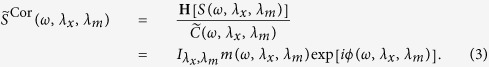


The decay frequency response 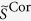 is ready to be analyzed by either iterative least square fitting with appropriate lifetime models, or a non-iterative complex phasor based approach we developed for real-time lifetime calculation^37^. The former method was used for analyzing all results in this paper. The latter method, which can be implemented in FPGA^37^, could allow real-time 3D lifetime image analysis in the future.

#### Volumetric imaging with SLOT

In order to obtain 3D spatial information of the sample, FmFLIM, the spectral measurement, was combined with SLOT, the spatial measurement. FmFLIM-SLOT data thus formed a 6-dimension volume 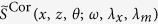, where *x, z, θ* are spatial coordinates of the tomographic projection scan.

To perform FmFLIM-SLOT, the modulated laser from the output of the interferometer was loosely focused to a 15-μm-wide beam with a depth of focus of more than 1 mm. The beam excited fluorophores along the line of laser path over the sample volume. Emission along the beam path was collected and recorded as a single pixel in the projection image (Supplementary Fig. S3). In this study, the combined power of multiple laser lines was typically between 0.1 to 0.5 mW. Because of the loose focus of the beam, the excitation intensity was much lower than confocal microscopy, and neither photobleaching of the fluorescent proteins nor phototoxicity was observed in live embryos. Fluorescent emission was collected at 90 degrees from the laser excitation line by a condenser lens (1 inch diameter, f = 30 mm) and a concave mirror (2 inch diameter, f = 25 mm). The total fluorescence collection solid angle was 1.32 sr, equivalent to the collection angle of an ideal 0.66 NA lens. Collimated fluorescent emissions were separated into multiple spectral bands by dichroic mirrors and bandpass filters and detected by multiple PMT detectors (Hamamatsu 7422). The transmitted excitation laser was collected by a photodiode detector to form transmission optical projection.

To take a FmFLIM projection data *S*(*x*, *z*, *θ*; *ω*, *λ*_*x*_, *λ*_*m*_) at a projection angle *θ*, two galvo mirrors scanned the focused laser line across the sample at a pixel size of 10 μm and a pixel rate of 44,000 pixels/sec. The sample was rotated in equal angle steps between each projection. A total of 180 projection frames were typically acquired with 2 degree angular steps. The size of the 2D projection frame was typically 200 × 350 pixels (x-by-z) in order to cover a whole zebrafish embryo. A complete projection dataset *S*(*x*, *z*, *θ*; *ω*, *λ*_*x*_, *λ*_*m*_) required 12 minutes of acquisition, in which approximately 5 minutes were spent on taking projection images and the rest on rotating the sample. In the future, data recording and sample rotating could be performed simultaneously by implementing spiral tomography, which could be cut down the acquisition time by half. The 6-D projection dataset *S*(*x*, *z*, *θ*; *ω*, *λ*_*x*_, *λ*_*m*_) was processed by MatLab scripts (MathWorks) to obtain the 3D multi-channel fluorescence lifetime images.

#### FmFLIM-SLOT data processing

Because the spatial dimensions (*x*, *z*, *θ*) and the time/spectral dimensions (*ω*, *λ*_*x*_, *λ*_*m*_) are orthogonal, time/spectral and spatial processing do not interfere with each other. Similar to single-channel FLIM tomography^11^,^12^, FmFLIM-SLOT data *S*(*x*, *z*, *θ*; *ω*, *λ*_*x*_, *λ*_*m*_) first underwent the spatial tomography reconstruction and was transformed to *S*(*x*, *z*, *y*; *ω*, *λ*_*x*_, *λ*_*m*_). Then, *S*(*x*, *z*, *y*; *ω*, *λ*_*x*_, *λ*_*m*_) was subjected to lifetime analysis on the spectral dimensions (Supplementary Fig. S18b).

The tomography reconstruction requires a known rotation center. The rotation center of the sample stage relative to the projection images was first measured with fluorescent beads phantom^40^ prior to *in vivo* imaging.

After tomography reconstruction, multi-channel decay data of reconstructed voxels were corrected for the system RF response as described previously according to Equations (1, 2, 3). Corrected decay data were analyzed with iterative least square fitting with frequency domain single exponential lifetime model, which yielded intensities *I*(*x*, *z*, *y*; *λ*_*x*_, *λ*_*m*_) and average lifetimes *τ*(*x*, *z*, *y*; *λ*_*x*_, *λ*_*m*_) at multiple Ex-Em channels. When the sample embryo contained a single FRET sensor, the donor channel lifetime image served as an indicator of the FRET efficiency. For dual FRET sensor imaging, in which two sensors had spectrally overlapping fluorophores, a combined intensity-lifetime analysis method was used to process triple-channel *I*(*x*, *z*, *y*; *λ*_*x*_, *λ*_*m*_) *τ*(*x*, *z*, *y*; *λ*_*x*_, *λ*_*m*_) volumes and to obtain individual readout for each FRET sensor (Supplementary Notes).

#### Rendering of 3D lifetime volumes

The 3D intensity and lifetime volumetric data were rendered into 2D projections for visualization. For intensity projections, 3D volumes of all spectral channels were rendered individually into 2D intensity projections by the maximum intensity projection (MIP) method, and then merged to false color 2D intensity projections. 2D Lifetime projections were rendered from 3D intensity-lifetime volumes through a modified MIP algorithm, which calculated lifetime projections as intensity-weighted averages of all layers and represented the average lifetime projection image on a false-color scale with the MIP intensity rendered as brightness.

All image reconstruction, data analysis and data visualization were performed using in-house software implemented in MatLab (Mathworks Inc.).

### Zebrafish Procedures

#### Zebrafish breeding and maintenance

Zebrafish (*Danio rerio*) were reared and maintained as described^41^. A lab-inbred AB* wild type strain was used for generating transgenic zebrafish. Embryos were collected after natural spawns, raised at 28.5 °C, and staged according to hours post fertilization (hpf) up to 10 hpf. The embryos were then kept at room temperature to slow development and prolong the imaging time window. Developmental stages of embryos were estimated by observation. After 20 hpf, embryos were transferred to embryo medium containing 0.2 mM 1-phenyl-2-thiourea to prevent pigmentation. All the animal procedures and experimental procedures were made in compliance with the “Guide for The Care and Use of Laboratory Animals (8^th^ Edition)”. The animal use protocol was approved by the University Committee on Use and Care of Animals at the University of Michigan (protocol #4478).

#### Injection of dyes for four-color zebrafish

Cy5-conjugated 500kDa dextran (0.5 mg/mL) was injected subcutaneously at the posterior trunk region of 72 hpf transgenic *Tg* (*enpep:GFP;pod:nfsB-mCherry*) zebrafish larvae. Following injection, the larvae were stained with 100 μM Syto 41 (Life Technologies) for 30 minutes^26^ and washed with embryo medium three times prior to imaging.

#### Cloning promoter and constructing plasmids

Promoters of *enpep* were amplified from zebrafish genomic DNA by PCR and cloned into the p5′E vector in the Tol2 gateway kit (generously provided by Drs. Chi-bin Chien and Kristen Kwan)^42^. The rtTA gene and the Tet-ON promoter (*P*_*Tight*_) were amplified from pRetroX-Tet-OnAdvanced and pRetroX-Tight-Pur (Clontech) and cloned into the pME and p5′E in the Tol2 gateway kit respectively. The GEpacmC sensor was a generous gift from Dr. Kees Jalink^43^. The CD2V Calcium sensor was obtained from Addgene (plasmid No. 37471).

The transgenic constructs were made according to the published procedure for the Tol2 gateway kit (http://tol2kit.genetics.utah.edu/index.php/Main_Page).

#### Generating transgenic zebrafish and microinjection

Capped RNA encoding the Tol2 transposase was synthesized from linearized plasmid using mMessage mMachine T3 RNA synthesis kit (Ambion) via *in vitro* transcription. To generate transgenic zebrafish, the DNA construct was co-injected with RNA encoding the Tol2 transposase into fertilized eggs. Embryos expressing the transgene were raised to adulthood and out-crossed to wild-type zebrafish to obtain germ-line transgenic animals. Sensor expression was induced by 10 μg/mL doxycycline (Sigma) treatment overnight. Since doxycycline has strong fluorescence under 405 nm excitation, embryos were soaked and washed in embryo medium for 30 minutes prior to imaging.

#### Generating Triple transgenic zebrafish with inducible expressions of CD2V and GEpacmC sensors

Transgenic zebrafish line *Tg*(*enpep:rtTA; P*_*Tight*_*:CD2V*) was crossed with transgenic zebrafish line *Tg*(*P*_*Tight*_*:GEpacmC*) to produce triple-transgenic offsprings with inducible expression of both CD2V and GEpacmC sensors in kidney tubules.

#### Zebrafish mounting

Zebrafish embryos were mounted following a multi-layer tube mounting protocol^44^ that is optimal for time lapse imaging of zebrafish embryos. Zebrafish embryos were placed in embryo medium containing 200 μg/mL tricaine and 0.1% low melting point agarose (Sigma) and then mounted inside 0.8 mm ID FEP plastic tubes (Cole Parmer) plugged with 1% agarose gel. The refractive index of the FEP plastic matches the index of water.

#### EGTA, BAPTA-AM and ionomycin treatment for zebrafish

Zebrafish embryos were treated for 2 hours in a mixture of calcium chelators EGTA (Sigma, 3mM), BAPTA-AM (Sigma, 100 μM) and the ionophore ionomycin (Millipore, 10 μM) prepared in embryo medium. The 2-hour treatment time ensured complete drug penetration over the entire embryo. For post treatment imaging, zebrafish embryos were mounted in the treatment solution with the addition of 0.1% low melting point agarose gel and 200 μg/mL tricaine. The treatment decreased Ca^2+^ level in the embryo younger than 36 hpf. Zebrafish embryos were washed and placed back into regular embryo medium after imaging. All embryos developed normally afterwards.

#### Forskolin and IBMX treatment for zebrafish

Zebrafish embryos were treated for 2 hours in a mixture of 100 μM adenylyl cyclase activator forskolin (Sigma) and 400 μM phosphodiesterase inhibitor IBMX (Sigma) in embryo medium. For post treatment imaging, zebrafish embryos were mounted in the treatment solution with the addition of 0.1% low melting point agarose gel and 200 μg/mL tricaine. The treatment increased cAMP level in the embryo. Zebrafish embryos were then washed and placed back into embryo medium. All embryos developed normally after the treatment.

## Additional Information

**How to cite this article**: Zhao, M. *et al.* Multiplexed 3D FRET imaging in deep tissue of live embryos. *Sci. Rep.* **5**, 13991; doi: 10.1038/srep13991 (2015).

## Supplementary Material

Supplementary Information

Supplementary Movie 1

Supplementary Movie 2

Supplementary Movie 3

Supplementary Movie 4

Supplementary Movie 5

Supplementary Movie 6

Supplementary Movie 7
