# Supplementary material for: Multiplexed 3D FRET imaging in deep tissue of live embryos
Source: Sci Rep. 2015 Sep 21;5:13991. doi: 10.1038/srep13991 (PMC4585674; doi:10.1038/srep13991)
Supplement: Supplementary Information [file srep13991-s1.pdf]

Scientific Reports

Multiplexed 3D FRET imaging in deep tissue of live embryos

Ming Zhao, Xiaoyang Wan, Yu Li, Weibin Zhou and Leilei Peng

Supplementary figures and notes

Supplementary Figure S1	*Volumetric imaging with scanning laser optical tomography (SLOT)*
Supplementary Figure S2	*Spatial resolution of SLOT*
Supplementary Figure S3	*Optical schematics and spectral configuration of the FmFLIM-SLOT system*
Supplementary Figure S4	*Pixel lifetime histograms of CD2V sensor in a Tg(enpep:rtTA; P_{Tight}:CD2V) embryo undergoing Ca^{2+} treatment*
Supplementary Figure S5	*Effect of Ca^{2+} treatment on CD2V sensor in multiple Tg(enpep:rtTA; P_{Tight}:CD2V) embryos between 30 and 36 hpf*
Supplementary Figure S6	*Ca^{2+} treatment lost effect on CD2V sensor in multiple Tg(enpep:rtTA; P_{Tight}:CD2V) embryos after 36 hpf*
Supplementary Figure S7	*Pixel lifetime histograms of GEpacmC sensor in a Tg(enpep:rtTA; P_{Tight}:GEpacmC) embryo undergoing cAMP treatment*
Supplementary Figure S8	*Effect of cAMP treatment on GEpacmC sensor in multiple Tg(enpep:rtTA; P_{Tight}:GEpacmC) embryos*
Supplementary Figure S9	*Ca^{2+} treatment did not affect GEpacmC sensor in multiple Tg(enpep:rtTA; P_{Tight}:GEpacmC) embryos*

Supplementary Figure S10	*cAMP treatment did not affect CD2V sensor in multiple Tg(enpep:rtTA;P_{Tight}:CD2V) embryos*
Supplementary Figure S11	*Pixel lifetime histograms of a Tg(enpep:rtTA;P_{Tight}:CD2V; P_{Tight}:GEpacmC) embryo undergoing Ca^{2+} treatment*
Supplementary Figure S12	*Pixel lifetime histograms of a Tg(enpep:rtTA;P_{Tight}:CD2V; P_{Tight}:GEpacmC) embryo undergoing cAMP treatment*
Supplementary Figure S13	*Pixel histograms of recovered GFP lifetime in a Tg(enpep:rtTA;P_{Tight}:CD2V; P_{Tight}:GEpacmC) embryo*
Supplementary Figure S14	*Effect of Ca^{2+} treatment on multiple Tg(enpep:rtTA; P_{Tight}:CD2V; P_{Tight}:GEpacmC) embryos*
Supplementary Figure S15	*Effect of cAMP treatment on multiple Tg(enpep:rtTA; P_{Tight}:CD2V; P_{Tight}:GEpacmC) embryos*
Supplementary Figure S16	*GFP lifetime pixel histogram of Tg(kdrl:GFP) embryo, showing the lifetime accuracy of FmFLIM-SLOT*
Supplementary Figure S17	*Modulation frequency sweeping in FmFLIM*
Supplementary Figure S18	*Data acquisition and analysis of FmFLIM-SLOT*
Supplementary Notes	*Combined intensity-lifetime analysis of dual FRET sensors*

Supplementary Movies:

Movie 1 Volumetric lifetime images of a *Tg* (*kdrl:GFP;pod:nfsB-mCherry*) embryo at 72 hpf. Left: false color intensity projection with GFP in green and mCherry in red. Middle: false color lifetime projection of GFP. Right: false color lifetime projection of mCherry. In false color lifetime projections, lifetime was rendered as color according to the color index on the right, and intensity was rendered as brightness.

Movie 2 Cross-section flythrough of the *Tg* (*kdrl:GFP; pod:nfsB-mCherry*) embryo. Left: false color intensity cross-section with GFP in green and mCherry in red. Middle: lifetime cross-section of GFP. Right: lifetime images of mCherry.

Movie 3 Four-channel volumetric lifetime images of *Tg*(*enpep:GFP; pod:nfsB-mCherry*) embryo with GFP (488-green channel) in kidney tubules, mCherry (561-red channel) in renal glomeruli, Cy5-conjugated dextran (640-deep red channel) in blood vessels via injection, and Syto 41 nuclear label (Invitrogen, 405-blue channel) stain in sensory neurons. Left: false color intensity projection with Syto 41 in blue, GFP in green, mCherry in red and Cy5 in white. Four panels to the right: lifetime projections of Syto 41, GFP, mCherry and Cy5 respectively.

Movie 4 A *Tg*(*enpep:rtTA; P_{Tight}:CD2V*) embryo before (top row) and after (bottom row) being treated with 3 mM EGTA, 100 μM BAPTA-AM and 10 μM ionomycin to decrease Ca^{2+} level. Left: false color intensity projection with CFP in blue and Venus in green. Right: CFP lifetime projection.

Movie 5 A *Tg*(*enpep:rtTA; P_{Tight}:GEpacmC*) embryo before (top row) and after (bottom row) being treated with 100 μM forskolin and 400 μM IBMX to increase cAMP level. Left: false color intensity projection with GFP in green and mCherry in red. Right: GFP lifetime projection.

Movie 6 A *Tg*(*enpep:rtTA; P_{Tight}:CD2V; P_{Tight}:GEpacmC*) embryo before (top row) and after (bottom row) being treated with 3 mM EGTA, 100 μM BAPTA-AM and 10 μM ionomycin to decrease Ca^{2+} level. Left: false color intensity projection with 405-blue channel in blue, 488-green channel in green, and 561-red channel in red. Three panels to the right: lifetime projection of 405-blue channel, 488-green channel and recovered GFP lifetime.

Movie 7 A *Tg*(*enpep:rtTA; P_{Tight}:CD2V; P_{Tight}:GEpacmC*) embryo before (top) and after (bottom) being treated with 100 μM forskolin and 400 μM IBMX to increase cAMP level. Left: false color intensity projection with 405-blue channel in blue, 488-green channel in green, and 561-red channel in red.

Three panels to the right: lifetime projections of 405-blue channel, 488-green channel and recovered GFP lifetime respectively.

Supplementary Figure S1 Volumetric imaging with scanning laser optical tomography (SLOT)

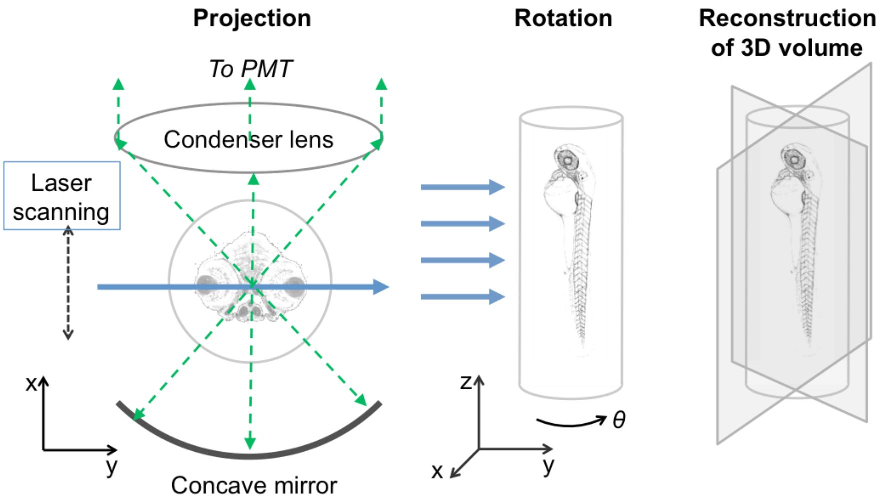

Fig. S1. Volumetric imaging with scanning laser optical tomography (SLOT). A loosely focused laser beam penetrates through the sample and excites fluorophores along its path. All fluorescent emission along the laser path is collected as a single pixel measurement by a concave mirror paired with a condenser lens. The laser is scanned across the sample to form a x-z plane projection. The sample is rotated around the z-axis between scans. Multiple projections are collected at different angles. The 3D volumetric image is reconstructed from 2D projections via inverse Radon transform.

Supplementary Figure S2   Spatial resolution of SLOT

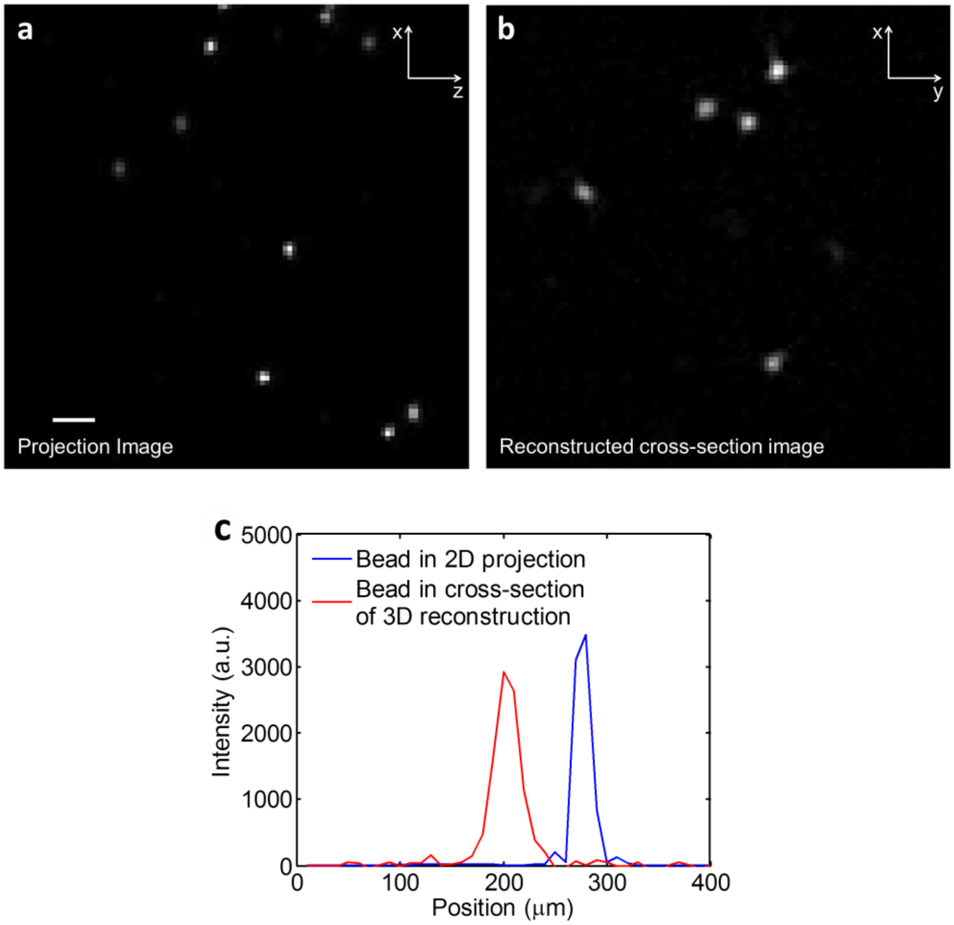

Fig. S2. Spatial resolution of SLOT, calibrated by imaging 2.5 μm diameter fluorescent beads (Invitrogen InSpeck Green) embedded in 1% agarose gel inside a 0.8 mm ID FEP tube. (a) x-z projection image of beads. (b) x-y cross-section image of the reconstructed 3D tomography volume. (c) Cross profile of a single bead in the 2D projection and the cross-section of reconstructed 3D volume. The resolution of the reconstructed 3D volume is slightly degraded by the filtered back projection algorithm of inverse Radon transform. The spatial resolution of the reconstructed 3D volume is 25 μm, determined by the FWHM of the bead cross section. Scale bar is 100 μm.

Supplementary Figure S3 Optical schematics and spectral configuration of the FmFLIM-SLOT system

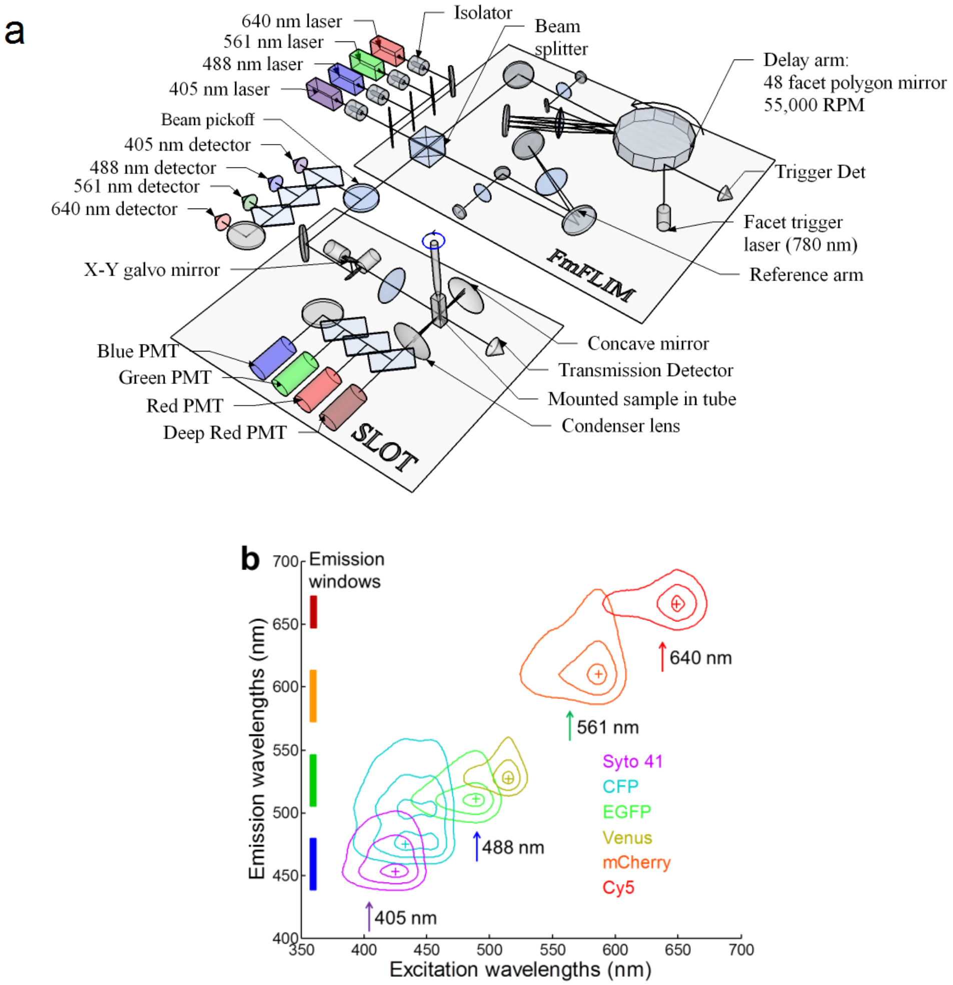

Fig. S3. Optical schematics and spectral configuration of the FmFLIM-SLOT system. (a) Optical setup[1]. Four excitation lasers (405 nm, 488 nm, 561 nm and 640 nm) are modulated by a Michelson interferometer with a spinning polygon mirror optical delay line. Modulation frequencies are inversely proportional to laser wavelengths. A beam pickoff at the output of the interferometer diverts a small portion of the multi-line laser output to a series of photodiodes, which monitor intensity modulations of individual laser lines. The output of the interferometer is focused to a 15-µm-wide beam with a depth of focus of more than 1 mm. The focused beam excites fluorophores along its path.

Fluorescent emission along the laser path is collected from the side by a condenser lens and a concave mirror, and detected by four PMT detectors designated for different emission spectral bands (Blue: 457±20 nm, Green: 525±22 nm, Red: 593±20 nm, Deep Red: 661±10 nm). Two galvo mirrors scan the focused laser line across the sample volume to obtain x-z fluorescence projections. The transmitted excitation laser is collected by a photodiode detector to form transmission optical projection. The sample is rotated and scanned at multiple angles. **b**. Spectral configuration of the FmFLIM-SLOT system, showing excitation-emission spectra of all fluorophores used in this study.

It is worth noting that the FmFLIM system can acquire all Ex-Em channels in parallel, which allows distinguishing between acceptor fluorescence due to FRET process and due to direct excitation of the acceptor by their different excitation sources. Large Stokes-shift signal from FRET-induced acceptor emission can be used in analyzing complex FRET phenomenon such as 3-color cascading FRET [2]. However in thick biological samples such as live zebrafish embryos, strong auto-fluorescence, which also tends to have a large Stokes-shift, coexists with acceptor fluorescence due to FRET. Thus it is highly challenging to make use of these large Stokes-shift channels.

Supplementary Figure S4 Pixel lifetime histograms of CD2V sensor in a Tg(enpep:rtTA; P_{Tight}:CD2V) embryo undergoing Ca^{2+} Treatment

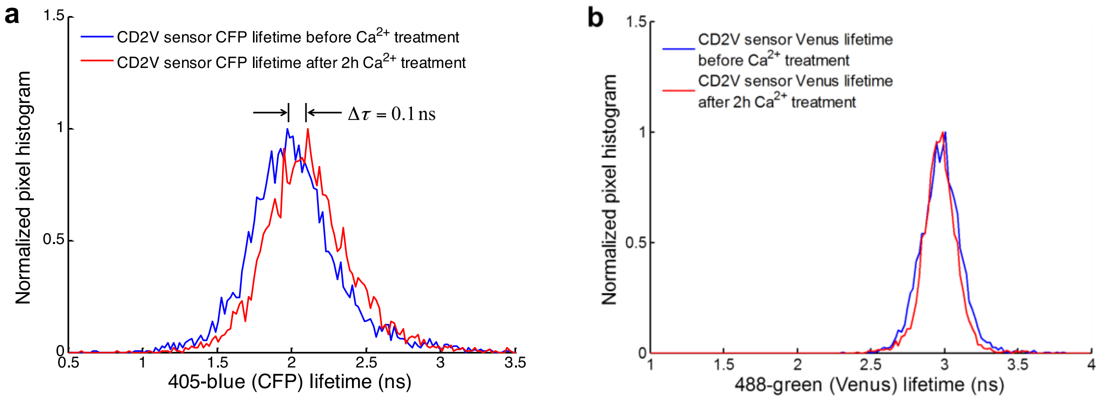

Fig. S4. Pixel lifetime histograms of CFP and Venus in a *Tg*(*enpep:rtTA; P_{Tight}:CD2V*) embryo undergoing Ca^{2+} treatment. (a) Pixel histogram of CFP (donor) lifetime measured in the 405-blue channel before and after a 2-hour treatment of 3 mM EGTA, 100 μM BAPTA-AM and 10 μM ionomycin. CFP lifetime showed a 0.1 ns increase due to the treatment. (b) Venus (acceptor) lifetime from its direct excitation-emission, measured in the 488-green channel, was not affected by the treatment and remained at 2.98 ns. The step size of histograms is 0.02 ns.

Supplementary Figure S5 *Effect of Ca^{2+} treatment on CD2V sensor in multiple Tg(enpep:rtTA; P_{Tight}:CD2V) embryos between 30 and 36 hpf*

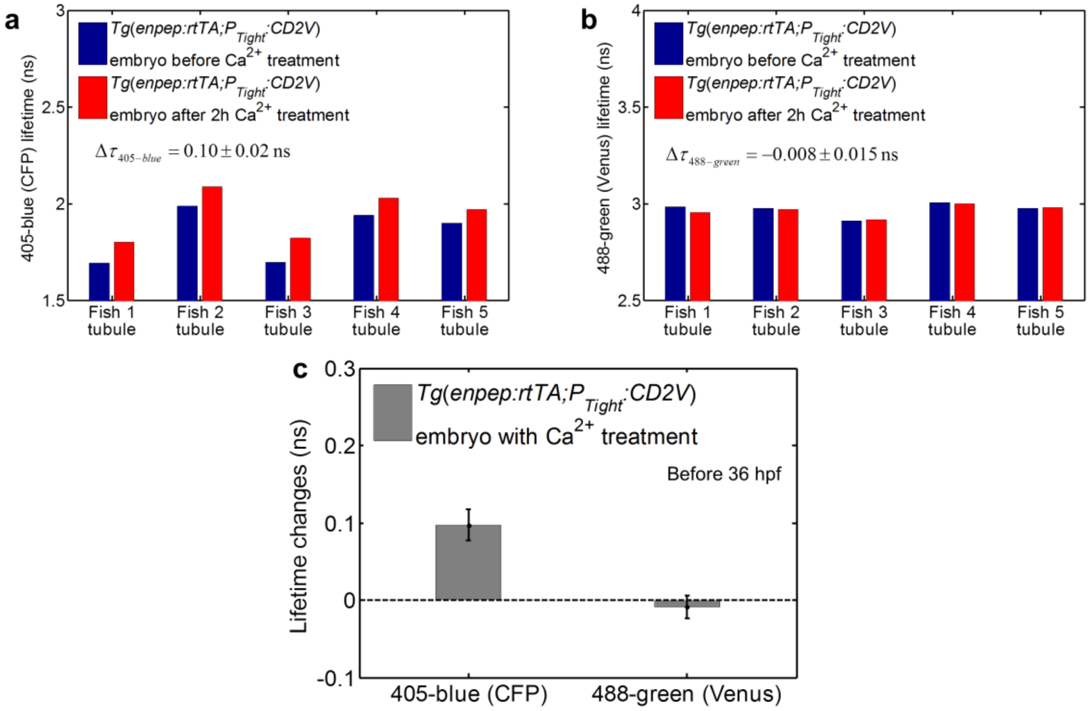

Fig. S5. Effect of Ca^{2+} treatment on CD2V sensor in multiple *Tg*(*enpep:rtTA; P_{Tight}:CD2V*) embryos between 30 and 36 hpf. (a) Average CFP (donor) lifetime (405-blue channel) before and after a 2-hour treatment of 3 mM EGTA, 100 µM BAPTA-AM and 10 µM ionomycin. The treatment increased CFP lifetime in embryos between 30 and 36 hpf, indicating decreased FRET efficiency and Ca^{2+} level. Increase in CFP lifetime (0.10±0.02 ns) was consistent over all embryos at. (b) Average Venus (acceptor) direct excitation-emission lifetime (488-green channel) was not affected by the Ca^{2+} treatment. (c) Statistical results from multiple embryos (N=5). Error bars are standard deviations of lifetime changes in multiple embryos.

Supplementary Figure S6 *Ca^{2+} treatment lost effect on CD2V sensor in multiple Tg(enpep:rtTA; P_{Tight}:CD2V) embryos after 36 hpf*

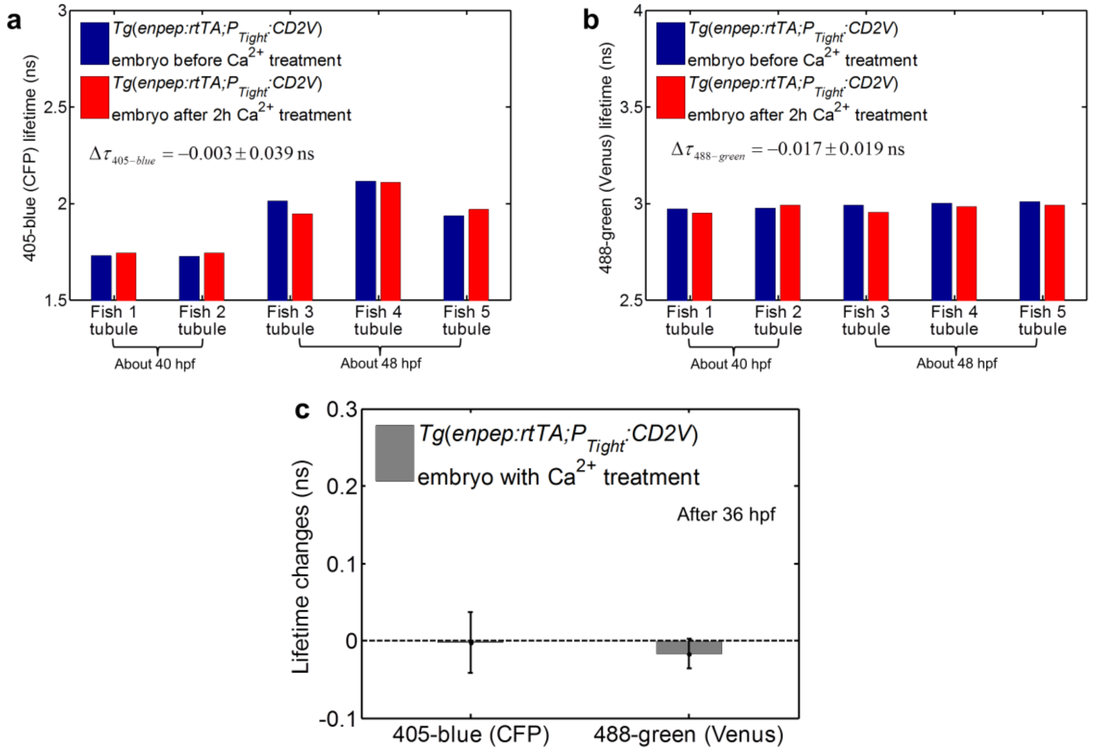

Fig. S6. Ca^{2+} treatment lost effect on CD2V sensor in multiple *Tg*(*enpep:rtTA; P_{Tight}:CD2V*) embryos older than 36 hpf. (a) Average CFP (donor) lifetime (405-blue channel) before and after a 2-hour treatment of 3 mM EGTA, 100 µM BAPTA-AM and 10 µM ionomycin. CFP lifetime did not change in embryos older than 36 hpf. (b) Venus (acceptor) direct excitation-emission lifetime (488-green channel) was not affected by the Ca^{2+} treatment. (c) Statistical results from multiple embryos (N=5). Error bars are standard deviations of lifetime changes in multiple embryos.

Supplementary Figure S7 Pixel lifetime histograms of GEpacmC sensor in a Tg(enpep:rtTA; P_{Tight}:GEpacmC) embryo undergoing cAMP treatment

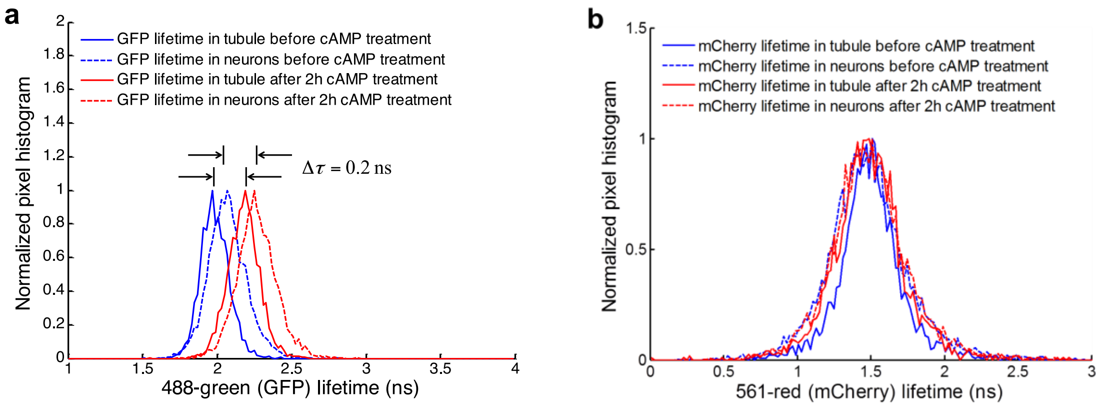

Fig. S7. Pixel lifetime histograms of GPF and mCherry in a *Tg*(*enpep:rtTA; P_{Tight}:GEpacmC*) embryo undergoing cAMP treatment. (a) Pixel lifetime histogram of GFP (donor) lifetime measured in 488-green channel. GFP lifetime in neurons was higher than in kidney tubules, suggesting higher cAMP level in neurons. After a 2-hour treatment of 100 μM forskolin and 400 μM IBMX for 2 hours, GFP lifetime showed a 0.2 ns increase in all tissue types. (b) mCherry (acceptor) direct excitation-emission lifetime, measured in 561-red channel, was not affected by the cAMP treatment in both tissue types. The step size of histograms is 0.02 ns.

Supplementary Figure S8 Effect of cAMP treatment on GEpacmC sensor in multiple Tg(enpep:rtTA; P_{Tight}:GEpacmC) embryos

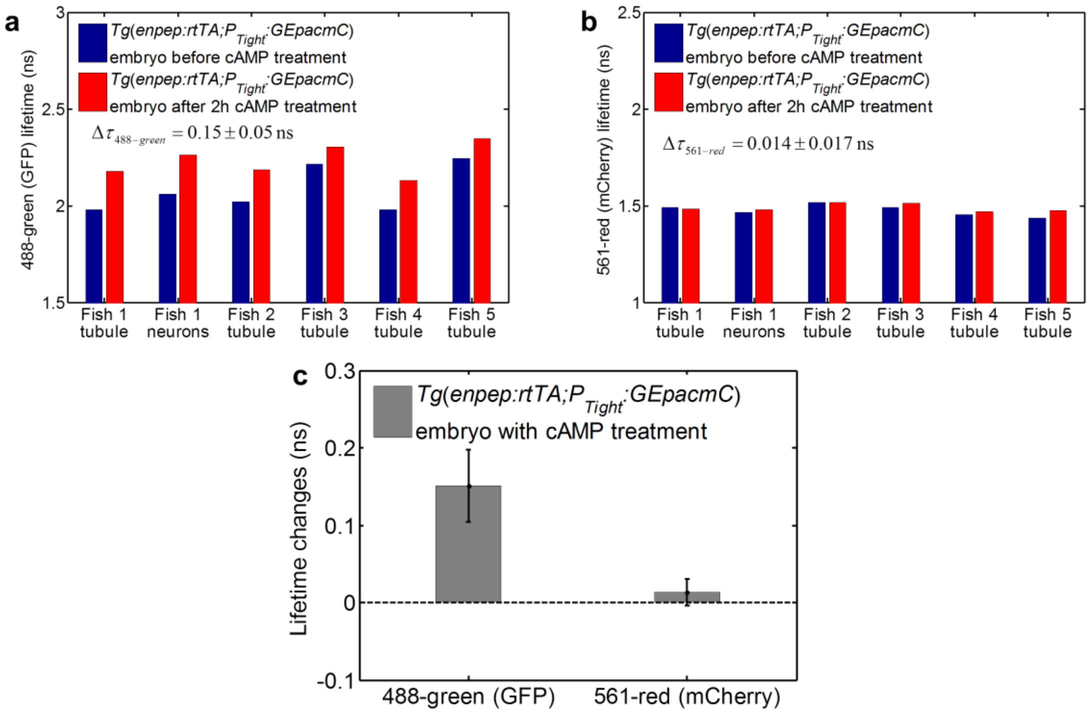

Fig. S8. Effect of cAMP treatment on GEpacmC sensor in multiple *Tg*(*enpep:rtTA; P_{Tight}:GEpacmC*) embryos between 40 and 48 hpf. (a) GFP (donor) lifetime (488-green channel) before and after cAMP treatment with 100 µM forskolin and 400 µM IBMX. The treatment increased GFP lifetime, indicating a decrease in FRET efficiency and increase in cAMP level. The increase in GFP lifetime (0.15±0.05 ns) was consistent in all embryos. (b) mCherry (acceptor) direct excitation-emission lifetime (561-red channel) was not affected by the cAMP treatment. (c) Statistical results from multiple embryos (N=5). Error bars are standard deviations of lifetime changes in multiple embryos.

Supplementary Figure S9 *Ca^{2+} treatment did not affect GEpacmC sensor in multiple Tg(enpep:rtTA; P_{Tight}:GEpacmC) embryos*

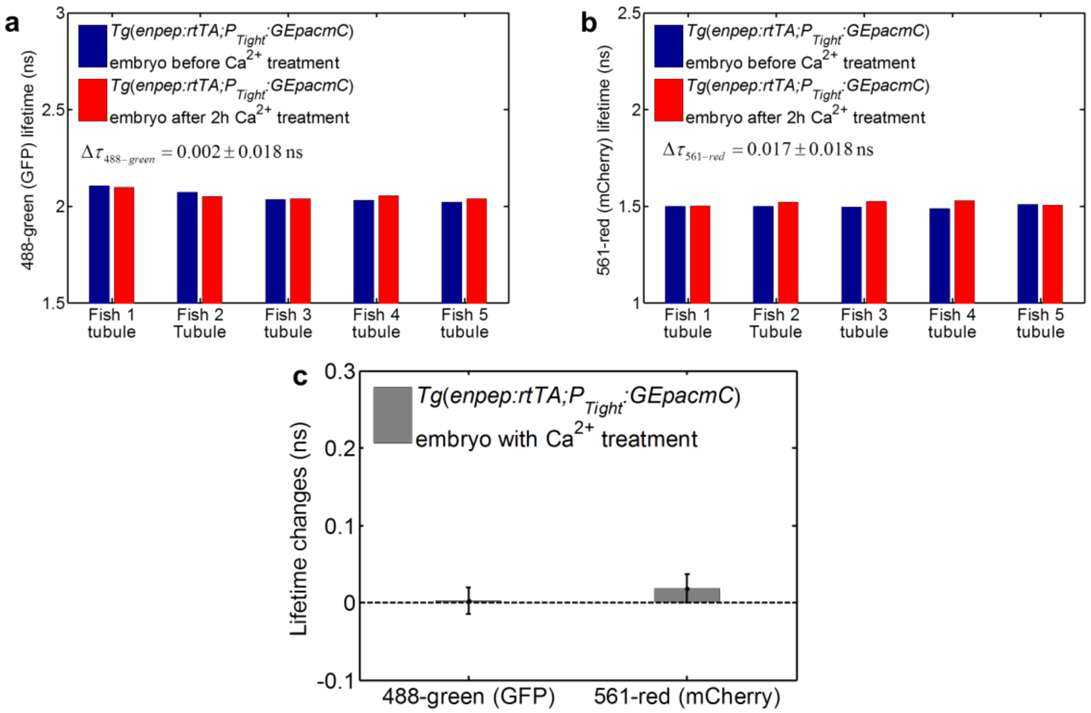

Fig. S9. Ca^{2+} treatment did not affect GEpacmC sensor in multiple *Tg*(*enpep:rtTA; P_{Tight}:GEpacmC*) embryos. (a) GFP (donor) lifetime (488-green channel) before and after a 2-hour treatment of 3 mM EGTA, 100 μM BAPTA-AM and 10 μM ionomycin. The treatment did not change GFP lifetime, indicating that the Ca^{2+} treatment had no effect on cAMP level. Embryos were between 30 and 36 hpf. (b) mCherry (acceptor) direct excitation-emission lifetime (561-red channel) was not affected by the Ca^{2+} treatment. (c) Statistical results from multiple embryos (N=5). Error bars are standard deviations of lifetime changes in multiple embryos.

Supplementary Figure S10 cAMP treatment did not affect CD2V sensor in multiple Tg(enpep:rtTA; P_{Tight}:CD2V) embryos

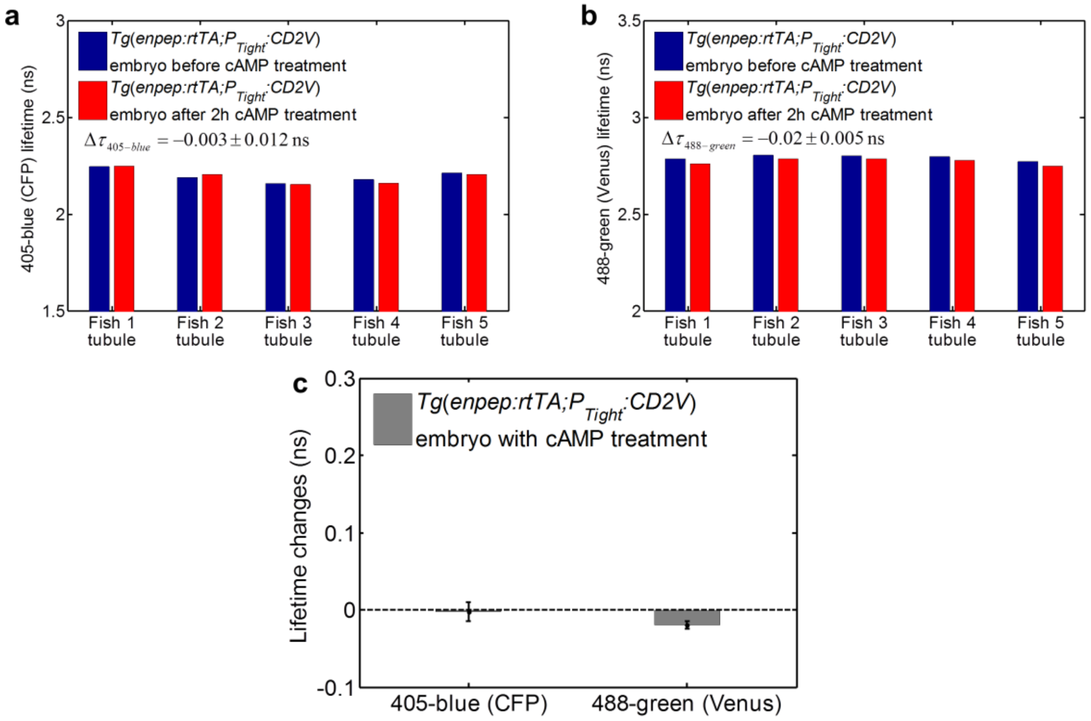

Fig. S10. cAMP treatment did not affect CD2V sensor in multiple *Tg*(*enpep:rtTA; P_{Tight}:CD2V*) embryos. (a) Donor CFP lifetime (405-blue channel) before and after cAMP treatment with 100 μM forskolin and 400 μM IBMX. The treatment did not change CFP lifetime, indicating that the cAMP treatment had no effect on Ca^{2+} level. (b) Venus (acceptor) direct excitation-emission lifetime (488-green channel) was not affected by the treatment. (c) Statistical results from multiple embryos (N=5). Error bars are standard deviations of lifetime changes in multiple embryos.

Supplementary Figure S11 Pixel lifetime histograms of a Tg(enpep:rtTA; P_{Tight}:CD2V; P_{Tight}:GEpacmC) embryo undergoing Ca^{2+} treatment

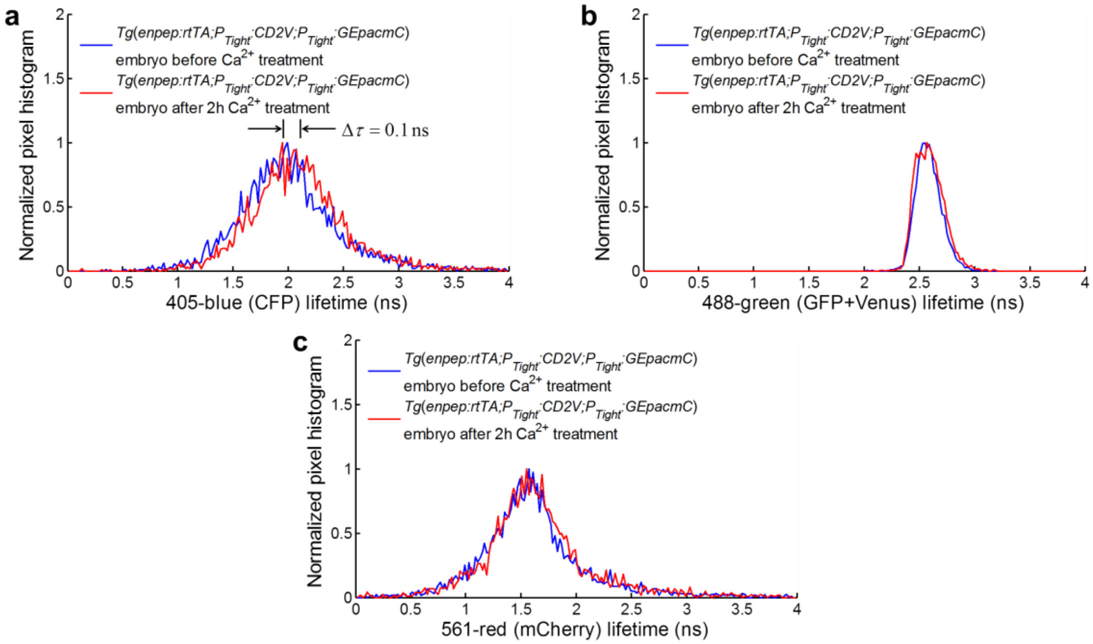

Fig. S11. Pixel lifetime histograms of three Ex-Ex channels from a *Tg*(*enpep:rtTA; P_{Tight}:CD2V; P_{Tight}:GEpacmC*) embryo undergoing Ca^{2+} treatment. (a) Pixel lifetime histograms of the 405-blue channel (CFP lifetime) before and after a 2-hour treatment of 3 mM EGTA, 100 μM BAPTA-AM and 10 μM ionomycin. CFP lifetime showed a 0.1 ns increase after the treatment. (b) Lifetime histogram of the 488-green channel (average lifetime of Venus and GFP) showed no significant change. (c) Lifetime histogram of the 561-red channel (mCherry lifetime) was not affected by the treatment. The step size of histograms is 0.02 ns.

Supplementary Figure S12 Pixel lifetime histograms of a Tg(enpep:rtTA; P_{Tight}*:CD2V;* P_{Tight}*:GEpacmC) embryo undergoing cAMP treatment*

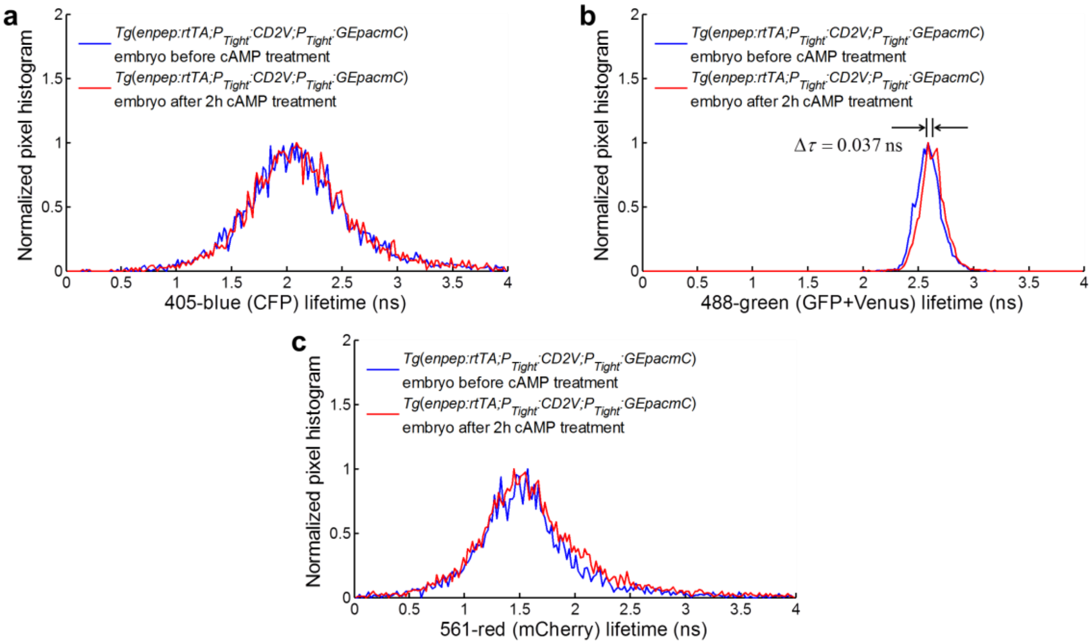

Fig. S12. Pixel lifetime histograms of three Ex-Ex channels from a *Tg*(*enpep:rtTA;*P_{Tight}*:CD2V;*P_{Tight}*:GEpacmC*) embryo undergoing cAMP treatment. (a) Lifetime histogram of the 405-blue channel (CFP lifetime) before and after a 2-hour treatment of 100 μM forskolin and 400 μM IBMX. CFP lifetime was not affected by the treatment. (c) Lifetime histogram of the 488-green channel (average lifetime of Venus and GFP) showed a small increase of 0.037 ns. (c) Lifetime histogram of the 561-red channel (mCherry lifetime) was not affected by the treatment. The step size of histograms is 0.02 ns.

Supplementary Fig. S13 *Pixel histograms of recovered GFP lifetime in a Tg(enpep:rtTA; P_{Tight}:CD2V; P_{Tight}:GEpacmC) embryo*

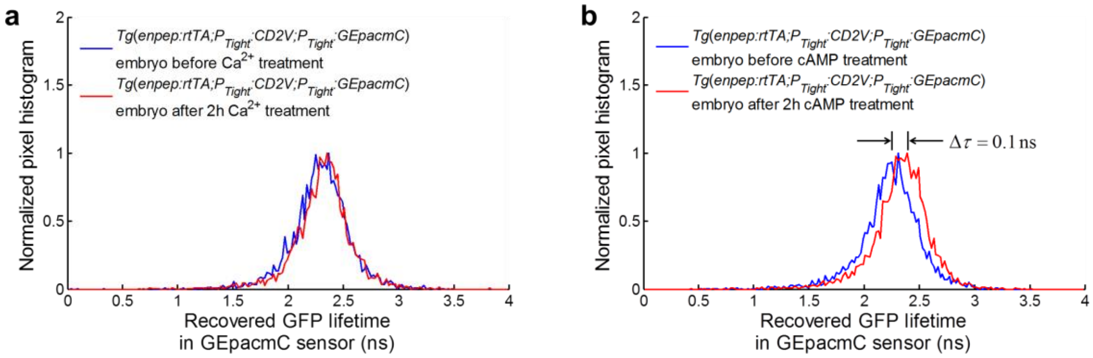

Fig. S13. Pixel histograms of recovered GFP lifetime in a *Tg*(*enpep:rtTA;P_{Tight}:CD2V;P_{Tight}:GEpacmC*) embryo. GFP lifetime was recovered from the 488-green channel lifetime data by the intensity-lifetime analysis method (see Suplementary Note). (a) The GFP lifetime of GEpacmC sensor showed no significant change after the Ca^{2+} treatment. (b) The recovered GFP lifetime of GEpacmC sensor was increased by 0.1 ns after the cAMP treatment. The step size of histograms is 0.02 ns.

Supplementary Fig. S14   *Effect of Ca^{2+} treatment on multiple $Tg(enpep:rtTA;P_{Tight}:CD2V; P_{Tight}:GEpacmC)$ embryos*

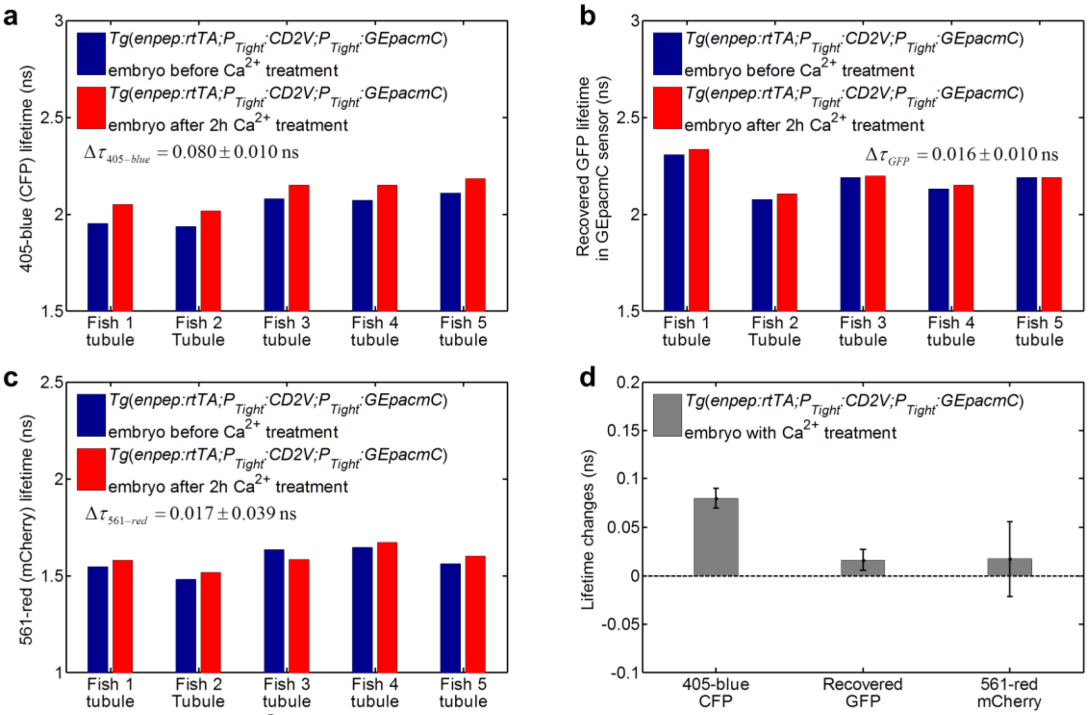

Fig. S14. Effect of Ca^{2+} treatment on multiple *Tg*(*enpep:rtTA;P_{Tight}:CD2V; P_{Tight}:GEpacmC*) embryos. (a) Lifetime of the 405-blue channel (CFP) before and after the Ca^{2+} treatment. CFP lifetime increased by 0.08±0.01 ns after the Ca^{2+} treatment. (b) GFP lifetime in GEpacmC sensor did not show significant change (0.016±0.010 ns). (c) Lifetimes in 561-red channel (mCherry) were not affected by the treatment (0.017±0.039 ns). (d) Statistical results from multiple embryos (N=5). Error bars are standard deviations of lifetime changes in multiple embryos.

Supplementary Fig. S15 *Effect of cAMP treatment on multiple Tg(enpep:rtTA;P_{Tight}:CD2V; P_{Tight}:GEpacmC) embryos*

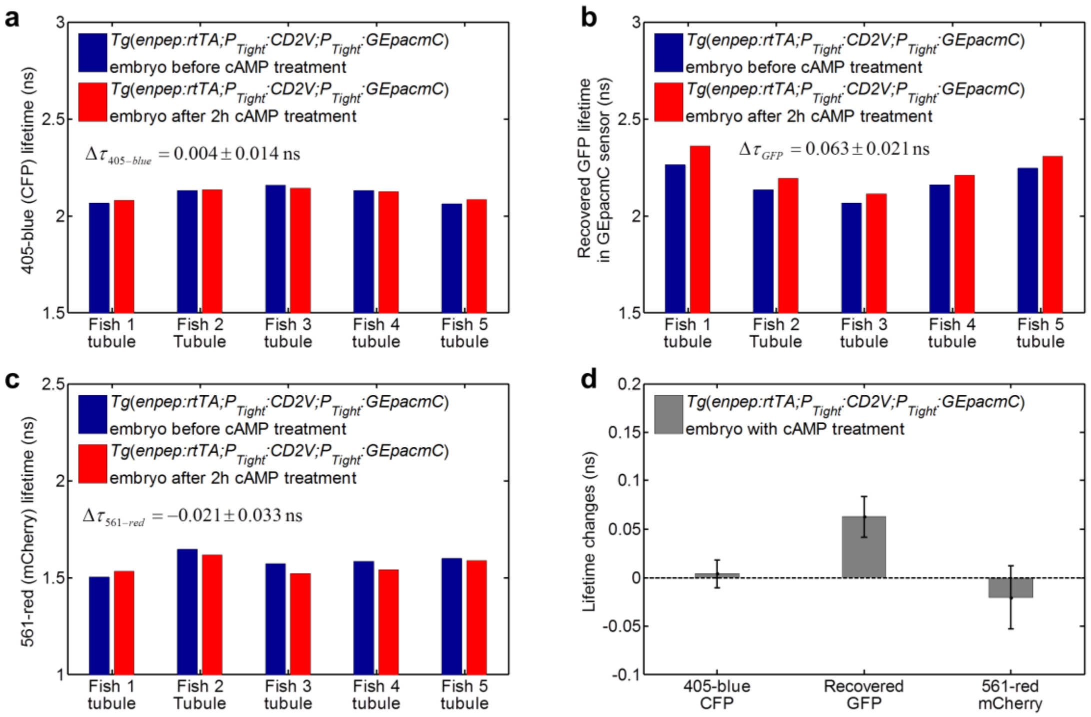

Fig. S15. Effect of cAMP treatment on multiple *Tg*(*enpep:rtTA;P_{Tight}:CD2V; P_{Tight}:GEpacmC*) embryos. (a) Lifetime of the 405-blue channel (CFP) was not affected by the cAMP treatment. (b) Recovered GFP lifetimes in GEpacmC sensors increased after the cAMP treatment. (c) Lifetime in the 561-red channel (mCherry) was not affected by the treatment (-0.02±0.03 ns). (d) Statistical results from multiple embryos (N=5). Error bars are standard deviations of lifetime changes in multiple embryos.

Supporting Figure S16 *GFP lifetime pixel histogram of Tg(kdrl:GFP) embryo, showing the lifetime accuracy of FmFLIM-SLOT*

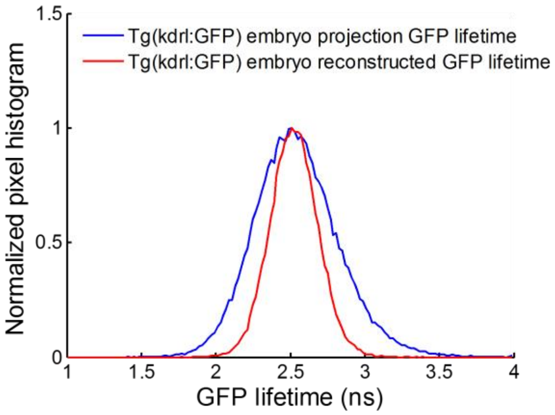

Fig. S16. GFP lifetime pixel histogram of *Tg*(*kdrl:GFP*) embryo, showing the lifetime accuracy of FmFLIM-SLOT. The 3D tomographic reconstruction averages over fluorescence photon signals from all projection angles, thus FmFLM-SLOT has a better lifetime accuracy than 2D projection FLIM. For *Tg*(*kdrl:GFP*) embryos, the lifetime accuracy of FmFLIM-SLOT is 2-times better than the accuracy of FmFLIM projection image (2.56±0.14 ns vs. 2.56±0.27 ns). The step size of the histogram is 0.02 ns.

Supplementary Fig. S17 Modulation frequency sweeping in FmFLIM

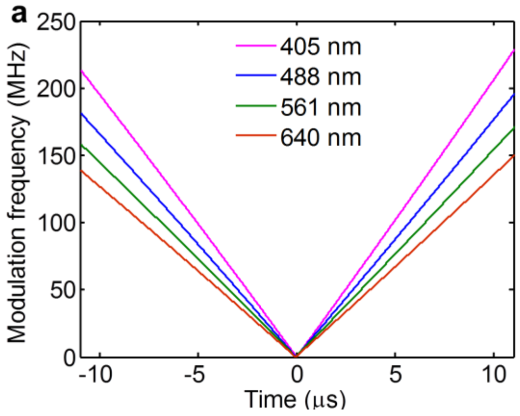

Fig. S17. Modulation frequency sweeping in FmFLIM. At the output of the Michelson interferometer, all laser lines are intensity-modulated into linear frequency sweeps. Instantaneous modulation frequencies are inversely proportional to laser wavelengths.

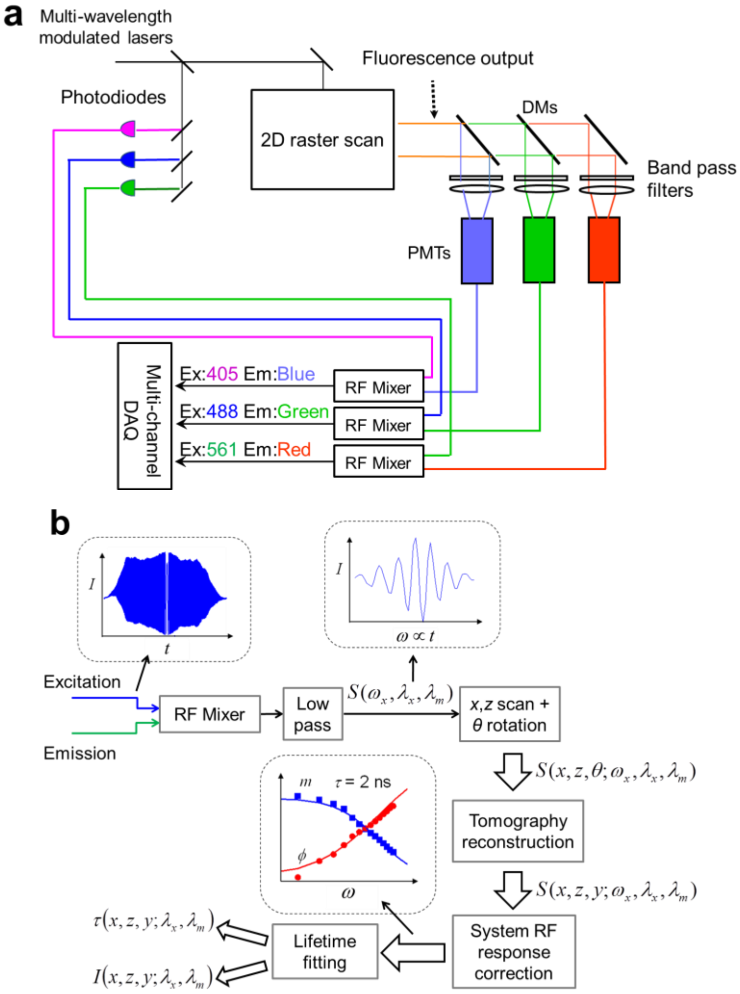

Fig. S18. Data acquisition and analysis of FmFLIM-SLOT. (a) The multi-wavelength modulated laser is scanned across the sample. Fluorescent photons are collected by multiple PMTs at different emission spectral bands. The modulation of each excitation laser line is monitored by a photodiodes. For each combination of Ex-Em wavelength, the corresponding laser modulation and the fluorescence signal are down-mixed with a RF analog mixer and collected by a multi-channel digitizer. DM: Dichroic mirror (b) high frequency excitation signal and fluorescence emission signal are down-mixed by the RF

mixer. The resulting low frequency signal carries lifetime information $S(\omega, \lambda_x, \lambda_m)$. After correcting for the system's RF response, the data is subjected to lifetime analysis.

Supplementary Notes

Combined intensity-lifetime analysis of dual FRET sensors

Four fluorescence proteins in the dual FRET sensor system with CD2V sensor and GEpacmC sensor are measured in three spectral channels: 405-blue (CFP), 488-green (Venus and GFP) and 561-red (mCherry). To quantify both FRET sensors, two donor lifetimes need to be extracted:

(1) the donor (CFP) lifetime of the CD2V sensor is directly measured in the 405-blue channel, not affected by the presence of the GEpacmC cAMP sensor;

(2) the donor (GFP) lifetime of the GEpacmC sensor can be indirectly measured through the 488-green channel, whose lifetime is an intensity weighted average of GFP lifetime in GEpacmC and Venus lifetime in CD2V,

$$\bar{\tau}_{Green}^{488} = \frac{I_{CD2V}^{Venus}\tau_{CD2V}^{Venus} + I_{GEpacmC}^{GFP}\tau_{GEpacmC}^{GFP}}{I_{GEpacmC}^{GFP} + I_{CD2V}^{Venus}}. \quad (1)$$

where I_{CD2V}^{Venus} and τ_{CD2V}^{Venus} are the intensity and lifetime of Venus in CD2V sensor; $I_{GEpacmC}^{GFP}$ and $t_{GEpacmC}^{GFP}$ are the intensity and lifetime of GFP in GEpacmC sensor. Since the lifetime of Venus is known and is not affected by FRET, if the relative intensity ratio between the two sensors is known, the GFP lifetime $\tau_{GEpacmC}^{GFP}$ can be calculated from Eq. 1.

In each FRET sensor used in this study, donors and acceptors are linked and expressed at a concentration ratio of 1:1, steady state fluorescence emission intensities of donors and acceptors are therefore related. For the CD2V sensor, the donor emission intensity is given by

$$I_{CD2V}^{CFP} = \eta^{CFP} \sigma^{CFP} I_{exc}^{CFP} C_{CD2V} \frac{\tau_{CD2V}^{CFP}}{\tau_0^{CFP}}, \quad (2)$$

where η^{CFP} is the combined quantum efficiency of fluorescence emission and detection system for CFP, σ^{CFP} is the excitation cross section of CFP, I_{exc}^{CFP} is the excitation power , C_{CD2V} is the concentration of the sensor, τ_{CD2V}^{CFP} is the lifetime of CFP in CD2V sensor, and τ_0^{CFP} is the baseline CFP lifetime without FRET. The acceptor emission intensity from direct excitation is similarly given by

$$I_{CD2V}^{Venus} = \eta^{Venus} \sigma^{Venus} I_{exc}^{Venus} C_{CD2V}, \quad (3)$$

where η^{Venus} is the combined quantum efficiency of fluorescence emission and detection system for Venus, σ^{Venus} is the excitation cross section of Venus, and I_{exc}^{Venus} is the excitation power for Venus. The emission intensity ratio between CFP and Venus is therefore

$$R_{CD2V} = \frac{I_{CD2V}^{CFP}}{I_{CD2V}^{Venus}} = \frac{\eta^{CFP} \sigma^{CFP} I_{exc}^{CFP}}{\eta^{Venus} \sigma^{Venus} I_{exc}^{Venus}} \frac{\tau_{CD2V}^{CFP}}{\tau_0^{CFP}} = K_{CD2V} \frac{\tau_{CD2V}^{CFP}}{\tau_0^{CFP}}. \quad (4)$$

The coefficient K_{CD2V} can be calibrated with embryos expressing CD2V sensor. Similarly for the GEpacmC sensor, the fluorescence emission ratio between GFP and mCherry is given by

$$R_{GEpacmC} = \frac{I_{GEpacmC}^{GFP}}{I_{GEpacmC}^{mCh}} = \frac{\eta^{GFP} \sigma^{GFP} I_{exc}^{GFP}}{\eta^{mCh} \sigma^{mCh} I_{exc}^{mCh}} \frac{\tau_{GEpacmC}^{GFP}}{\tau_0^{GFP}} = K_{GEpacmC} \frac{\tau_{GEpacmC}^{GFP}}{\tau_0^{GFP}}, \quad (5)$$

where η^{GFP} and η^{mCh} are combined quantum efficiencies of fluorescence emission and detection system for GFP and mCherry respectively, σ^{GFP} and σ^{mCh} are the excitation

cross sections, I_{exc}^{GFP} and I_{exc}^{mCh} are excitation powers of GFP and mCherry respectively, $\tau_{GEpacmC}^{GFP}$ is the lifetime of GFP in GEpacmC sensor, and τ_0^{GFP} is the baseline GFP lifetime without FRET. The coefficient $K_{GEpacmC}$ can be calibrated from embryos expressing cAMP sensors.

With intensity ratios between donor and acceptor known for the two sensors, GFP and Venus fluorescence intensities in Eq. 1 can be calculated from CFP and mCherry intensities

$$I_{CD2V}^{Venus} = \frac{I_{CD2V}^{CFP}}{R_{CD2V}} = \frac{\tau_0^{CFP} I_{CD2V}^{CFP}}{\tau_{CD2V}^{CFP} K_{CD2V}}, \qquad (6)$$

and

$$I_{GEpacmC}^{GFP} = R_{GEpacmC} I_{GEpacmC}^{mCh} = K_{GEpacmC} \frac{\tau_{GEpacmC}^{GFP}}{\tau_0^{GFP}} I_{GEpacmC}^{mCh}. \qquad (7)$$

In practice, because the 561-red channel that measures mCherry generally have less tissue autofluorescence and better signal-to-noise than the 405-blue channel that measures CFP, we used mCherry intensity measured by the 561-red channel to calculate $I_{GEpacmC}^{GFP}$, and use the relation

$$I_{green}^{488} = I_{GEpacmC}^{GFP} + I_{CD2V}^{Venus}. \qquad (8)$$

to obtain Venus intensity I_{CD2V}^{Venus}. By bringing in Eqs. 7 and 8, Eq.1 becomes

$$\bar{\tau}_{green}^{488} = \tau_{CD2V}^{Venus} + \frac{K_{GEpacmC} I_{GEpacmC}^{mCh}}{I_{green}^{488} \tau_0^{GFP}} \tau_{GEpacmC}^{GFP} \left(\tau_{GEpacmC}^{GFP} - \tau_{CD2V}^{Venus} \right) \qquad (9)$$

The GFP lifetime $\tau_{GEpacmC}^{GFP}$ is calculated by solving Eq. 9.

1. Zhao, M., Y. Li, and L. Peng, *Parallel excitation-emission multiplexed fluorescence lifetime confocal microscopy for live cell imaging.* Optics Express, 2014. **22**(9): p. 10221-10232.
2. Zhao, M., R. Huang, and L. Peng, *Quantitative multi-color FRET measurements by Fourier lifetime excitation-emission matrix spectroscopy.* Optics Express, 2012. **20**(24): p. 26806-27.
